# A T Cell-Inducing Influenza Vaccine for the Elderly: Safety and Immunogenicity of MVA-NP+M1 in Adults Aged over 50 Years

**DOI:** 10.1371/journal.pone.0048322

**Published:** 2012-10-31

**Authors:** Richard D. Antrobus, Patrick J. Lillie, Tamara K. Berthoud, Alexandra J. Spencer, James E. McLaren, Kristin Ladell, Teresa Lambe, Anita Milicic, David A. Price, Adrian V. S. Hill, Sarah C. Gilbert

**Affiliations:** 1 The Jenner Institute, University of Oxford, Oxford, United Kingdom; 2 Institute of Infection and Immunity, Cardiff University School of Medicine, Cardiff, United Kingdom; Statens Serum Institute, Denmark

## Abstract

**Background:**

Current influenza vaccines have reduced immunogenicity and are of uncertain efficacy in older adults. We assessed the safety and immunogenicity of MVA-NP+M1, a viral-vectored influenza vaccine designed to boost memory T cell responses, in a group of older adults.

**Methods:**

Thirty volunteers (aged 50–85) received a single intramuscular injection of MVA-NP+M1 at a dose of 1·5×10^8^ plaque forming units (pfu). Safety and immunogenicity were assessed over a period of one year. The frequency of T cells specific for nucleoprotein (NP) and matrix protein 1 (M1) was determined by interferon-gamma (IFN-γ) ELISpot, and their phenotypic and functional properties were characterized by polychromatic flow cytometry. In a subset of M1-specific CD8^+^ T cells, T cell receptor (TCR) gene expression was evaluated using an unbiased molecular approach.

**Results:**

Vaccination with MVA-NP+M1 was well tolerated. ELISpot responses were boosted significantly above baseline following vaccination. Increases were detected in both CD4^+^ and CD8^+^ T cell subsets. Clonality studies indicated that MVA-NP+M1 expanded pre-existing memory CD8^+^ T cells, which displayed a predominant CD27^+^CD45RO^+^CD57^−^CCR7^−^ phenotype both before and after vaccination.

**Conclusions:**

MVA-NP+M1 is safe and immunogenic in older adults. Unlike seasonal influenza vaccination, the immune responses generated by MVA-NP+M1 are similar between younger and older individuals. A T cell-inducing vaccine such as MVA-NP+M1 may therefore provide a way to circumvent the immunosenescence that impairs routine influenza vaccination.

**Trial Registration:**

ClinicalTrials.gov NCT00942071

## Introduction

Winter epidemics of influenza in the UK have caused 7,000–25,000 deaths in the past decade (1999–2010) [1]. In addition, influenza infection exerts pressure on healthcare systems and results in substantial economic losses. The burden of disease in developed countries disproportionately affects the elderly, with approximately 90% of influenza-associated excess deaths occurring among people aged 65 years and older [2]. Indeed, in those over the age of 75 years, 2·5–8·1% of all deaths in the UK in the last decade have been attributed to influenza virus infection [1].

Government-funded vaccination programmes for influenza exist in many countries and include elderly individuals in their target populations [3]. Unfortunately, the rates of seroprotection and seroconversion following vaccination are significantly lower in the elderly [4]. A recent systematic review found vaccine efficacy was 59% in adults aged under 65 years, but no trials assessing protection from laboratory-confirmed influenza have been conducted in subjects aged over 65 years [5].

In the elderly, immunosenescence can negatively impact the ability of the immune system to mount an effective immune response to new pathogens and vaccines. Characteristics of immunosenescence include: (i) a decrease in B cell function, which is thought to result from defective T cell help; (ii) thymic involution and an associated reduction in naive T cell output; (iii) expansion of selected memory T cell clones driven by persistent viral infections such as CMV (reported to affect up to 90% of elderly individuals) [6], and; (iv) increases in anergic CD28^−^ T cells and regulatory T cells [7]. Accordingly, there is an urgent need for an effective influenza vaccine targeted to the requirements of the ageing immune system.

In addition to seasonal epidemics, influenza can cause pandemics, typically following a viral antigenic shift. Therefore, new vaccine candidates should ideally induce an element of cross-strain (heterosubtypic) immunity [8]. One approach is to generate high frequencies of CD8^+^ T cells directed against conserved influenza antigens [9]. Viral-vectored vaccines elicit potent T cell responses and therefore represent a promising strategy in this regard. Modified Vaccinia virus Ankara (MVA) is a highly attenuated strain of vaccinia virus in which viral replication is blocked at a late stage of virion assembly [10]. Recombinant MVAs are therefore efficient single-round expression vectors, and have been used to prime or boost T cell responses to a diverse range of pathogen-specific and tumour-derived antigens. Previously, we have shown that a recombinant MVA expressing the nucleoprotein (NP) and matrix protein 1 (M1) sequences from a H3N2 strain of influenza A (termed MVA-NP+M1), was safe and immunogenic in young adults, significantly boosted T cell responses to NP and M1 [11] and has a protective effect against influenza challenge [12]. Such an approach may help to circumvent the limitations of immunosenescence, by boosting pre-existing memory T cell responses rather than by attempting *de novo* priming from the naïve lymphocyte pool. We have now extended the Phase I trial into older adults, and demonstrate here that MVA-NP+M1 is safe and highly immunogenic in this population.

## Methods

### Study Design

This was a Phase I open-label, non-randomized vaccine trial. The study was conducted at the Centre for Clinical Vaccinology and Tropical Medicine, University of Oxford, Oxford, UK. The clinical trial protocol and supporting CONSORT checklist are available as Supplementary Information; see Protocol S1 and Checklist S1. The trial protocol was approved within the UK by the Medicines and Healthcare products Regulatory Agency and the Gene Therapy Advisory Committee. The stated objectives of the trial were to assess the safety and the cellular immune response of a new influenza vaccine, MVA-NP+M1, when administered to healthy volunteers. The trial was registered at www.clinicaltrials.gov (identifier: NCT00942071).

**Figure 1:**
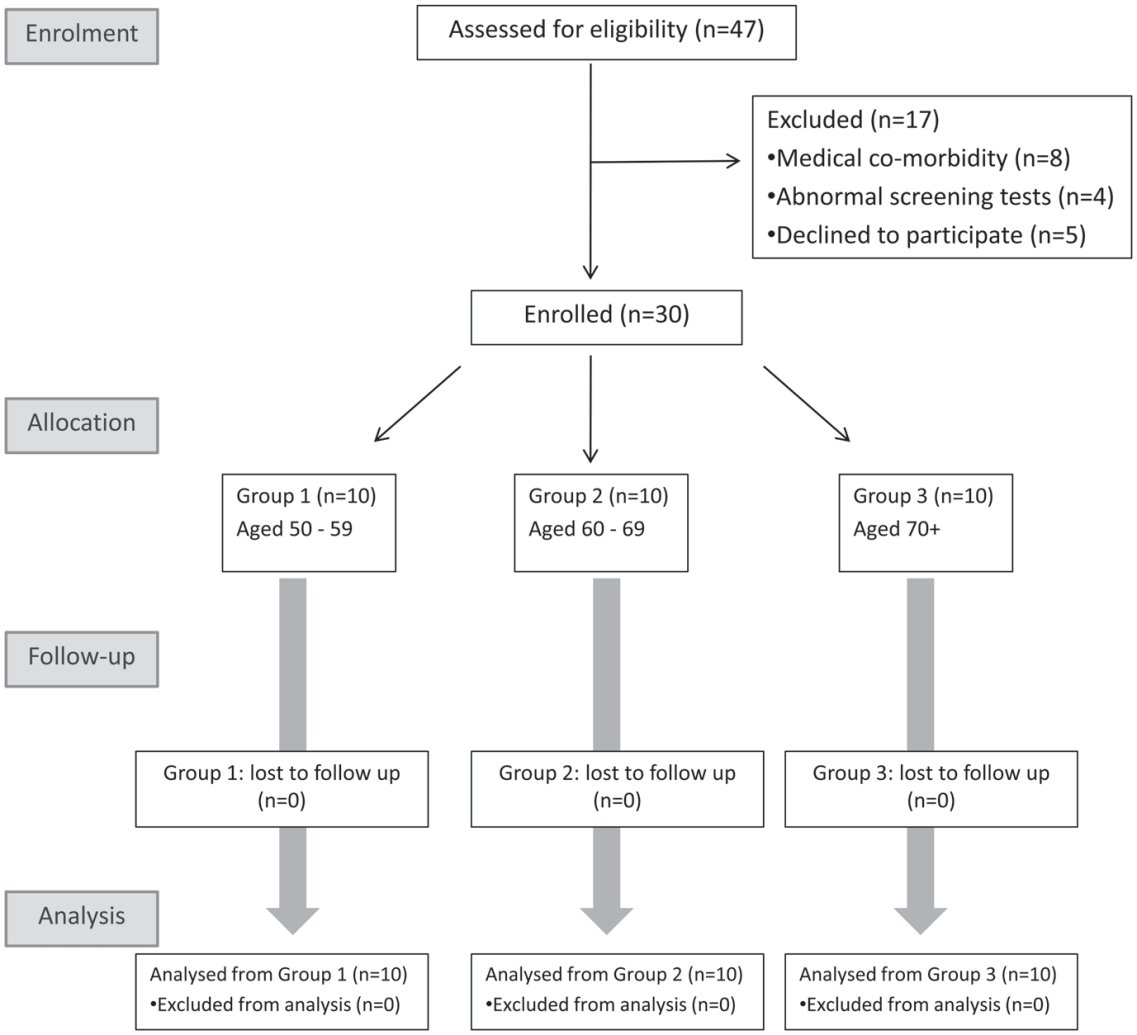
CONSORT flow diagram of the trial.

**Figure 2:**
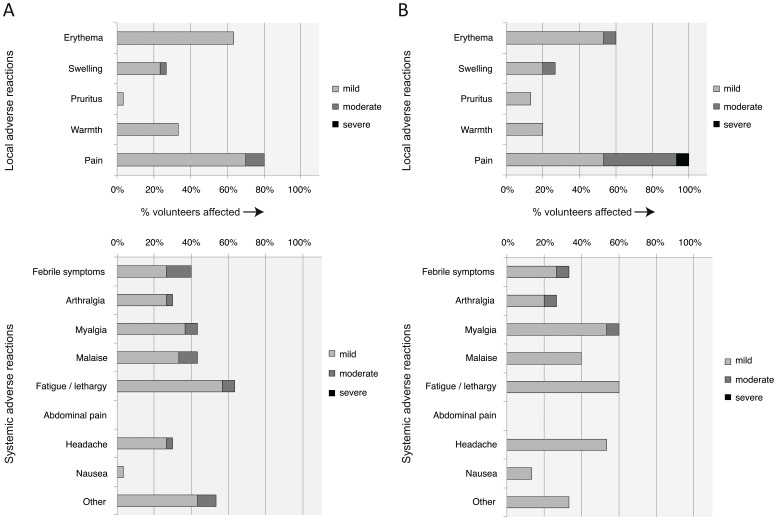
Frequency of local and systemic adverse events that were possibly, probably or definitely related to vaccination. (A) Volunteers aged 50+ (n = 30). (B) Volunteers aged 18–45 (n = 15). For both age groups pain was the most frequently recorded local adverse event followed by erythema. A similar pattern of systemic adverse events was observed in both age groups with the majority of solicited adverse events occurring in 20–60% of individuals. For volunteers aged 18–45, 85% of adverse events were mild; for volunteers aged 50+, 87% of adverse events were mild.

**Table 1:** Demographic characteristics of volunteers vaccinated in each cohort.

Group	Age range (years)	Mean age(years)	Females
**50–59**	50–59	55·2	50%
**60–69**	60–66	63·3	70%
**70+**	72–85	79·0	50%

### Participants

Thirty subjects were enrolled in three stratified age groups: 50–59 years, 60–69 years and 70+ years (10 volunteers per group). Younger volunteers from our previous two clinical trials were used for comparative purposes [11], [12]. All volunteers were healthy adults, resident in the Oxford area, with negative pre-vaccination tests for HIV antibodies, hepatitis B surface antigen and hepatitis C antibodies (see Supplementary Information: Protocol S1 for the full list of inclusion and exclusion criteria). Written informed consent was obtained in all cases. The planned sample size was 10 in each age group. This sample size should allow determination of the magnitude of the outcome measures, especially of serious and severe adverse events, rather than aiming to obtain statistical significance.

**Figure 3:**
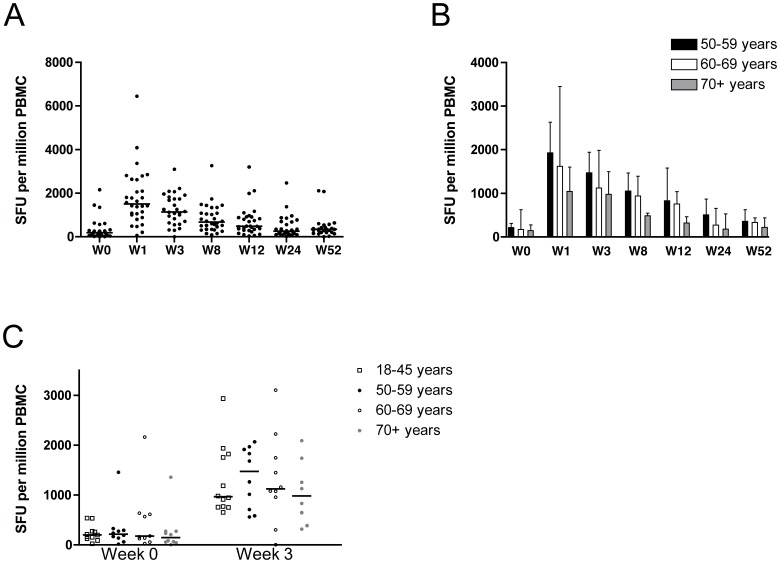
Ex vivo IFN-γ ELISpot responses to the vaccine insert. (A) Median and individual ex vivo IFN-γ ELISpot responses from vaccinated volunteers at baseline (week 0), and weeks 1, 3, 8, 12, 24, and 52. Significant differences between the pre- and post-vaccination time points were detected using the Wilcoxon signed rank test: week 1 (p = 0·0001), week 3 (p = 0·0001), week 8 (p = 0·0001), and week 12 (p = 0·001). (B) Median *ex vivo* IFN-γ ELISpot responses to the NP+M1 insert stratified according to age: black bars  =  group 1 (50–59 years), white bars  =  group 2 (60–69 years), and grey bars  =  group 3 (70+ years). Error bars indicate interquartile ranges. Significant differences between the pre- and post-vaccination time points were detected using the Wilcoxon signed rank test as follows. Group 1: week 1 (p = 0·002), week 3 (p = 0·002), week 8 (p = 0·002), week 12 (p = 0·039), week 24 (p = 0·002), and week 52 (p = 0·0039). Group 2: week 1 (p = 0·002), week 3 (p = 0·002), week 8 (p = 0·002), and week 12 (p = 0·0371). Group 3: week 1 (p = 0·0039) and week 3 (p = 0·0195). Significant differences were also detected between groups using the Mann-Whitney U-test, with responses in group 1 being higher than those in group 3 at week 3 (p = 0·043) and week 8 (p = 0·023). (C) Median and individual *ex vivo* IFN-γ ELISpot responses at week 1 and week 3 stratified according to age, and including a vaccinated cohort of younger (18–45 years) volunteers.

### MVA-NP+M1 Vaccine

The vaccine was described previously and consists of MVA expressing the NP and M1 antigens from influenza A as a single fusion protein [11].

**Figure 4:**
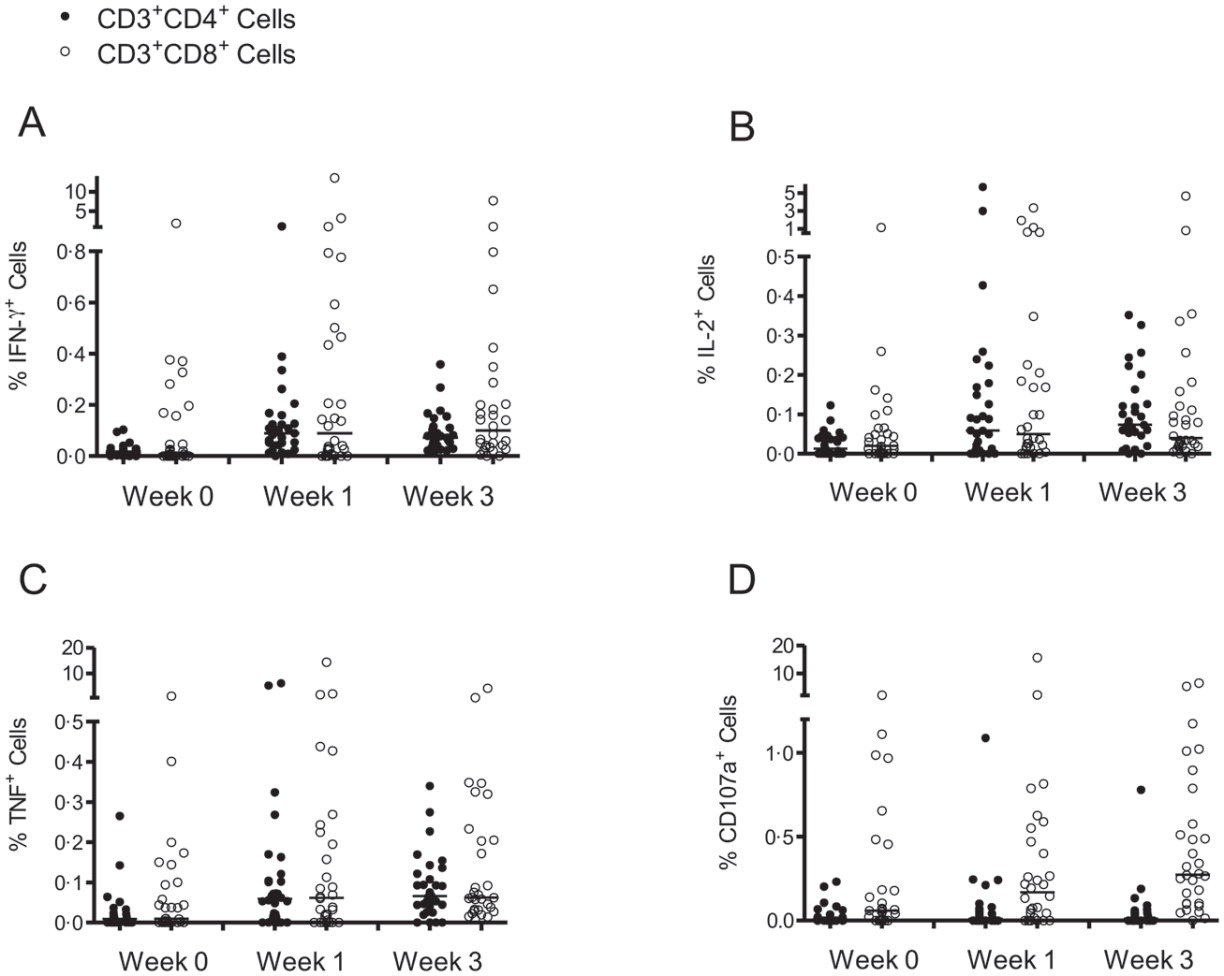
IFN-γ, IL-2, TNF and CD107a responses to the vaccine insert measured by flow cytometry. Production of IFN-γ (A), IL-2 (B) and TNF (C), and mobilization of CD107a (D), after background subtraction in CD3^+^CD4^+^ (black circles) and CD3^+^CD8^+^ (white circles) cell populations stimulated with a single pool of peptides spanning the complete NP+M1 vaccine insert. Volunteers in group 3 were tested at weeks 0, 1, and 3. Significant differences between pre- and post-vaccination time points were detected using the Wilcoxon signed rank test as follows: IFN-γ CD4^+^, week 1 (p = 0·0001) and week 3 (p = 0·0001); IFN-γ CD8^+^, week 1 (p = 0·001) and week 3 (p = 0·0005); IL-2 CD4^+^, week 1 (p = 0.001) and week 3 (p = 0·0001); IL-2 CD8^+^, week 1 (p = 0·006) and week 3 (p = 0·03); TNF CD4^+^, week 1 (p = 0·002) and week 3 (p = 0·0003); TNF CD8^+^, week 1 (p = 0·0003) and week 3 (p = 0·002); CD107a CD8^+^, week 3 (p = 0·004).

### Procedures

Volunteers were vaccinated on the day of enrolment with a single intramuscular injection of MVA-NP+M1 at a dose of 1·5×10^8^ pfu into the deltoid region of the arm. Blood was taken prior to the vaccination (week 0), and volunteers were observed for a period of 1 hour following the vaccination. Volunteers were given a digital thermometer, tape measure and symptom diary card to record their daily temperature, injection site reactions and solicited adverse events for 7 days. Two days after vaccination, volunteers were reviewed in clinic and assessed for potential adverse events. Volunteers were reassessed and blood samples were taken at subsequent visits, which occurred at 1, 3, 8, 12, 24, and 52 weeks post-vaccination.

**Figure 5:**
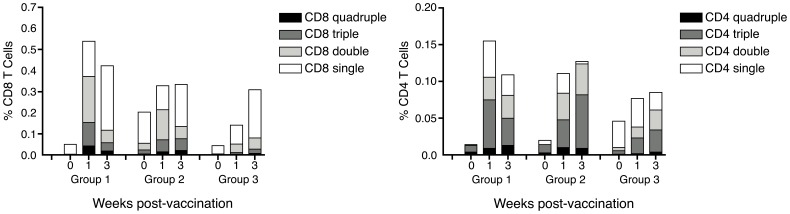
Functional profile of T cell responses to the vaccine insert measured by flow cytometry. Mobilization of CD107a and production of IFN-γ, IL-2 and TNF after background subtraction in CD3^+^CD4^+^ (A) and CD3^+^CD8^+^ (B) cell populations stimulated with a single pool of peptides spanning the complete NP+M1 vaccine insert. Median percentages of quadruple (black), triple (dark grey), double (light grey) and single (white) functional cells are shown.

### Interferon-gamma ELISpot

*Ex vivo* interferon-gamma enzyme-linked immunosorbent spot (IFN-γ ELISpot) assays were performed using fresh peripheral blood mononuclear cells (PBMC) as described previously [13]. Cells were washed and resuspended in RPMI 1640 containing 10% fetal calf serum, 100 IU/mL penicillin, 100 µg/mL streptomycin (all Sigma), and 2 mM L-glutamine (Life Technologies) (R10 medium). Peptides of 15–20 amino acids in length, overlapping by 10 amino acids and spanning the whole of the NP+M1 insert, were used to stimulate PBMC at a final concentration of 10 µg/ml in 8 pools of 10 peptides. R10 medium alone was used as a negative control, and a mixture of phytohaemagglutinin (PHA; 10 µg/mL) and staphylococcal enterotoxin B (SEB; 1 µg/mL) was used as a positive control. Each condition was assayed in triplicate using 2×10^5^ PBMC in a final volume of 100 µl per well. ELISpot plates were incubated for 18–20 hours at 37°C. Developed and dried ELISpot plates were analysed with an AID ELISpot reader (AID Diagnostika). Results are expressed as spot-forming units (SFU) per million PBMC, calculated by subtracting the mean R10 negative control response from the mean peptide pool response and summing the net response for the 8 peptide pools. Plates were excluded if responses were greater than 100 SFU/million PBMC in the R10 wells or less than 1,000 SFU/million PBMC in the PHA/SEB wells.

**Figure 6:**
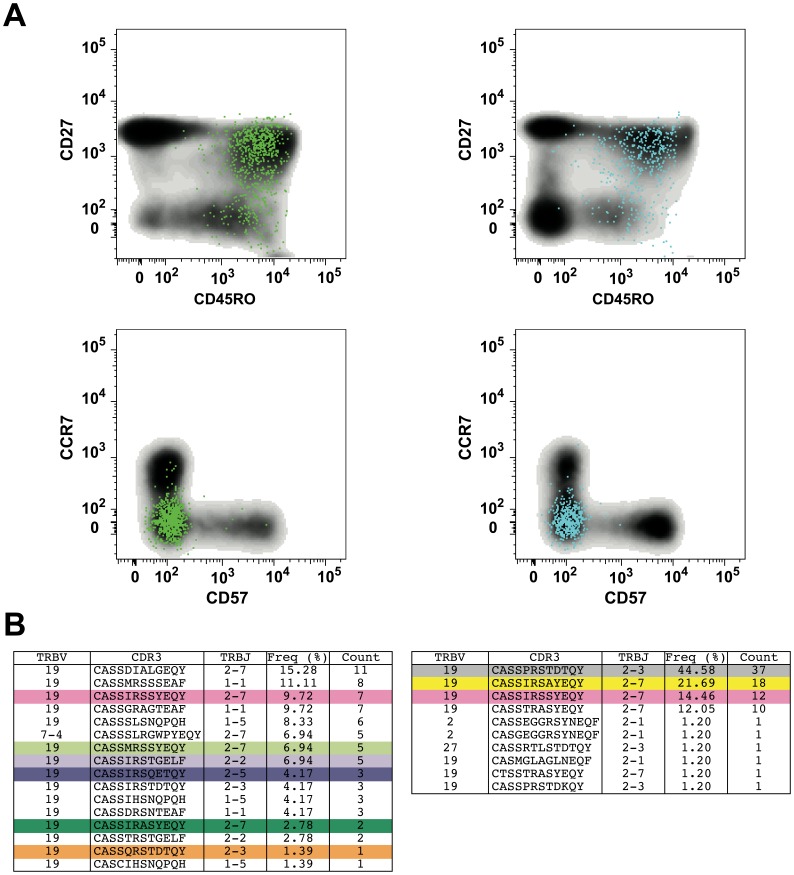
Phenotypic and clonotypic properties of M1-specific CD8^+^ T cells elicited by MVA-NP+M1. (A) Phenotype of vaccine-elicited CD8^+^ T cells specific for the HLA A*0201-restricted M1-derived epitope GILGFVFTL (residues 58–66). Antigen-specific CD3^+^CD8^+^tetramer^+^ cells are shown as coloured dots superimposed on bivariate plots showing the phenotypic distribution of the total CD8^+^ T cell population (grey density plots). Response sizes were 1·48% (left panels) and 0·75% (right panels) with respect to the total CD8^+^ T cell population. (B) TRBV and TRBJ usage, CDR3 amino acid sequence and relative frequency of the GILGFVFTL-specific CD8^+^ T cell clonotypes contained within the antigen-specific populations depicted in (A). Public clonotypes within the present dataset are colour-coded. Representative analyses are shown for volunteers in group 3 (70+ years).

**Figure 7:**
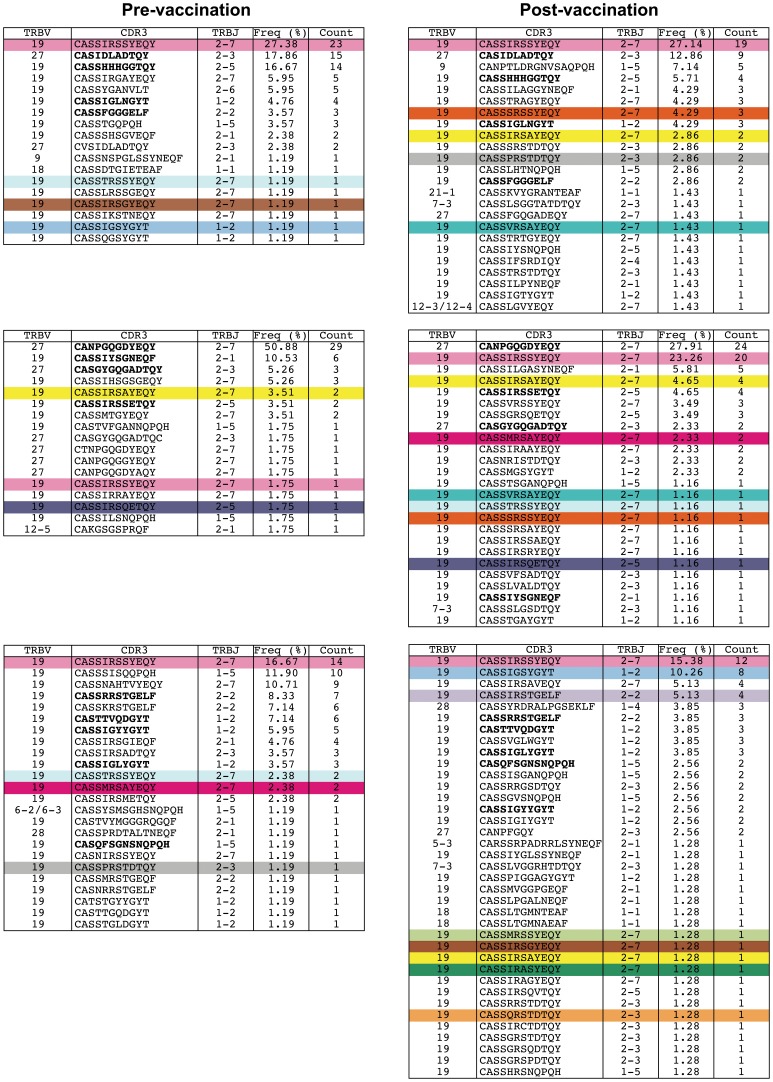
Patterns of clonotype usage in M1-specific CD8^+^ T cell populations before and after vaccination with MVA-NP+M1. TRBV and TRBJ usage, CDR3 amino acid sequence and relative frequency are shown for GILGFVFTL-specific CD8^+^ T cell clonotypes on day 0 (pre-vaccination) and day 7 (post-vaccination). Public clonotypes within the present dataset are colour-coded. Non-public clonotypes present at both time points within an individual are highlighted in bold type.

### Intracellular Cytokine Staining

Intracellular cytokine staining (ICS) was performed using two different T cell staining panels at week 0, week 1 and week 3. The first panel detected Th1-type cytokine (IFN-γ, IL-2 and TNF) production and CD107a mobilization. The second panel detected CD107a mobilization, granzyme B expression, and the production of IL-10 and IL-17. Reagent details are provided in Table S1.

Fresh PBMC (1–2×10^6^) were stimulated for 18 hr at 37°C with either a single pool comprising of all the NP+M1 peptides at a final concentration of 4 µg/ml, SEB (1 µg/ml) or medium alone. The costimulatory monoclonal antibodies (mAbs) αCD28 and αCD49d (1 µg/ml each; BD Pharmingen) were added to panel 1, and αCD107a-PE-Cy5 (10 µl; eBioscience) was added to both panels. After 2 hr, brefeldin A and monensin (both eBioscience) were added. The cells were then washed, stained with the reagents listed in Table S1 according to standard procedures and acquired using an LSR II flow cytometer (BD Biosciences). Data were analysed using FlowJo version 9·4 (Tree Star, Inc.). Unstained cells and compensation beads (BD Biosciences) stained singly with the individual mAbs in each panel were used as controls to calculate compensation. All mAbs were titrated for optimal staining. Between 13,000 and 700,000 live, CD14^−^CD19^−^ lymphocyte events were collected and analysed per condition.

### TCR Clonotyping

Cryopreserved PBMC were thawed and labelled with PE-conjugated GILGFVFTL/HLA A*0201 tetramer as described previously [14], then washed and surface stained with the directly conjugated mAbs listed in Table S1. Dead cells were excluded using LIVE/DEAD® Fixable Violet (Life Technologies), together with CD14^+^ and CD19^+^ events, in a single “dump” channel. Viable CD3^+^CD8^+^tetramer^+^ cells (918–5,000 per population) were sorted at >98% purity using a customized FACSAria II flow cytometer (BD Biosciences) and clonotypic analysis was conducted using a template-switch anchored RT-PCR as described previously [14], [15]. The IMGT nomenclature was used to assign *TRB* gene usage [16].

### Statistical Analysis

Statistical analysis was carried out using GraphPad Prism software version 5·04. The non-parametric Mann-Whitney U-test was employed to test for significant differences between groups of volunteers, and the non-parametric Wilcoxon signed rank test was used to test for significant differences between time points within the same group of volunteers.

## Results

Thirty volunteers were enrolled between Apr 06, 2010 and Nov 30, 2011 (Figure 1 and Table 1). Vaccination was well tolerated and no serious vaccine-related clinical or laboratory adverse events were observed. The frequency of local and systemic adverse reactions is shown in Figure 2. All vaccine-related adverse events were either mild or moderate in severity.

Figure 3a shows the T cell responses to the NP+M1 vaccine insert as measured by IFN-γ ELISpot. All assays were conducted on fresh PBMCs as soon as they became available. As expected, T cell responses to NP and M1 were detected prior to vaccination with a median response of 188 SFU/million PBMC. These responses increased to a median of 1,603 SFU/million PBMC one week after vaccination, representing an 8.5-fold increase. When the data were stratified for age (group 1 = 50–59 years, group 2 = 60–69 years, group 3 = 70+ years), differences between the groups became apparent. In particular, T cell responses to NP and M1 remained significantly above baseline until week 52 for group 1, week 12 for group 2 and week 3 for group 3 (Figure 3b).

We previously vaccinated 15 healthy volunteers aged 18–45 years with MVA-NP+M1 using the same dose (1·5×10^8^ pfu) and route of administration [12]. No significant differences in the ELISpot responses were detected between the younger volunteers (18–45 years) and the older volunteers (50+ years) either before or after vaccination (Figure 3c), although there was a trend towards higher responses in groups 1 and 2, and lower responses in group 3.

Flow cytometry was used to determine antigen-specific cytokine production (IFN-γ, TNF, IL-2, IL-10 and IL-17), T cell degranulation (CD107a mobilization) and granzyme B expression. MVA-NP+M1 was shown to boost both CD4^+^ and CD8^+^ T cell responses, and significant increases in IFN-γ, IL-2 and TNF production were observed in both populations at week 1 and week 3 post-vaccination (Figure 4). A significant increase in CD107a mobilization was only detected for CD8^+^ T cells at week 3 (p = 0·004).

No increases in the antigen-specific production of IL-10, IL-17 or granzyme B were detected in either the CD4^+^ or CD8^+^ T cell populations following vaccination (data not shown). However, as reported previously [17] the production of granzyme B from unstimulated CD8^+^ T cells was significantly elevated at week 1 in the oldest age group (median = 57·8% of CD8^+^ T cells) compared to group 1 (median = 27·1%, p = 0.002) and group 2 (median = 29·3%, p = 0.0115). Non-specific granzyme B production by CD8^+^ T cells was also significantly elevated in group 3 compared to group 1 at week 3 (group 3 = 45·9%, group 1 = 25·2%; p = 0.0113).

Figure 5 shows the frequency of polyfunctional CD4^+^ and CD8^+^ T cells detected by flow cytometry using panel 1. The percentage of T cells with quadruple, triple and double functional outputs detected in the CD4^+^ and CD8^+^ populations increased significantly at week 1 and week 3 post-vaccination in group 1 (50–59 years). However, in groups 2 and 3, only the triple and double functional cells in the CD4^+^ T cell population increased at the same time points. In group 3, a significant increase in quadruple and triple functional cells in the CD8^+^ T cell population was detected at week 3 post-vaccination. The respective P values for these comparisons (Wilcoxon signed rank test) are shown in Table S2.

In further analyses, we examined the clonotypic composition of CD8^+^ T cells specific for the HLA A*0201-restricted GILGFVFTL epitope (M1, residues 58–66) using a template-switch anchored RT-PCR to amplify all expressed *TRB* gene products. A profound type IV bias was observed in these antigen-specific CD8^+^ T cell populations, comprising strict TRBV19 usage combined with a central XRSX motif in the CDR3 loop (Figure 6) [18], consistent with previous reports [19], [20]. To determine the origins of these MVA-NP+M1 vaccine-expanded clonotypes, we conducted similar studies in a separate cohort of volunteers. These volunteers were aged 20–50 and had been vaccinated with either 5×10^7^ pfu intradermally or 2·5×10^8^ pfu intramuscularly [11]. Paired samples from day 0 (pre-vaccination) and day 7 (post-vaccination) were available for three volunteers. In all cases, the dominant clonotypes were identical at both time points, indicating the expansion of pre-existing M1-specific memory CD8^+^ T cells (Figure 7). However, the post-vaccination repertoires were more polyclonal due to the presence of less frequent clonotypes in greater numbers. This could reflect either *de novo* recruitment from the naïve pool or the expansion of clonotypes from the memory pool with pre-vaccination frequencies below the limit of detection. Notably, all sorted M1-specific CD8^+^ T cell populations displayed a predominant CD27^+^CD45RO^+^CD57^−^CCR7^−^ memory phenotype (Figure 6 and data not shown). This phenotypic homogeneity is consistent with the functional homogeneity observed within the CD8^+^ compartment before and after vaccination.

## Discussion

Here, we report the ability of MVA-NP+M1 to boost influenza-specific T cell responses in older adults. Recombinant MVA vaccines are establishing a good reputation for safety, although the majority of these data relate to younger individuals aged between 18–45 years. Our results with MVA-NP+M1 add to the experience from cancer trials with MVA-5T4 that recombinant MVA is safe in older adults [21]. Indeed, no severe or serious adverse reactions were detected in our volunteers.

We also report that MVA-NP+M1 is highly immunogenic in volunteers over the age of 50 years. In one quantitative review [4] of trivalent inactivated influenza vaccines, rates of seroprotection and seroconversion among those over 60 years old were four times lower for H1 and B antigens, and twice as low for H3 antigens. In addition, although not powered to detect declining efficacy with age, an age stratification suggested a far lower efficacy rate for those over 70 years [4]. Indeed, other studies have suggested that vaccine efficacy appears to be as low as 30–40% in this age group [22]. On an individual level, declines in immunological function are unlikely to occur in a linear fashion (chronological age being only a surrogate indicator of biological age) [23] However, on a population level, declines in vaccine responsiveness are likely to be observed as average age increases. Indeed, in the oldest age group (70+ years), we observed a reduction in immunogenicity as detected by *ex-vivo* IFN-γ ELISpot compared to the youngest age group (50–59 years), with significantly lower responses at 3 and 8 weeks post-vaccination. However, when the 3 age groups were compared to a younger cohort of volunteers (18–45 years) who received the same dose of the MVA-NP+M1 vaccine, no significant differences were detected.

The functional characteristics of the cellular responses produced by vaccination are potentially as important as magnitude [24]. Subsets of CD4^+^ and CD8^+^ T cells following vaccination with MVA-NP+M1 are capable of secreting both TNF and IL-2, in addition to IFN-γ. Increases in the number of such polyfunctional T cells have been associated with protective immunity in some models of infection [25]. We show here that MVA-NP+M1 vaccination can also induce polyfunctional CD4^+^ and CD8^+^ T cell responses in older adults, as determined by flow cytometric assessment of CD107a mobilization and the production of IFN-γ, TNF and IL-2.

MVA-NP+M1 is designed to expand T cells that are already present in the memory pool rather than prime naïve T cells *de novo*. Direct evidence for this mode of action comes from our comparison herein of M1-specific TCR sequences before and after vaccination. This provides a biological rationale for the use of MVA-NP+M1 in elderly individuals due to the impairment of thymic output with age. The absolute number of NP- and M1-specific T cells required for host defence against influenza is not known. However, the median *ex vivo* IFN-γ ELISpot response observed in the older volunteers peaked one week after vaccination at 1,603 SFU/million PBMC, which represents an 8·5-fold increase compared to the pre-vaccination response.

No vaccine-induced expression of granzyme B, IL-10 or IL-17 was detected in our cohort of older volunteers. However, we did detect significantly higher non-specific levels of granzyme B expression in group 3 (70+ years) compared to group 1 (50–59 years) at weeks 1 and 3 post-vaccination. It has been shown previously that baseline granzyme B expression in CD8^+^ T cells is higher in ageing volunteers and that these cells are associated with a decreased ability to respond to stimulation with whole influenza virus [17]. Degranulation and extracellular release of granzyme B can also cause inflammation and extracellular granzyme B has been implicated in increasing the risk of serious illness in the elderly, including the risk of influenza induced cardiovascular complications [26], [27].

A high IFN-γ:IL-10 ratio may be associated with protection from influenza [28]. The median frequency of NP- and M1-specific T cells that secreted IL-10 was low (below 0·006%) and did not increase after vaccination, whereas there was a significant increase in the number IFN-γ-secreting T cells following vaccination.

The memory phenotype of vaccine-induced CD8^+^ T cell populations, at least for a subset of M1-specific cells, was remarkably similar to that observed pre-vaccination. Indeed, a marginal decrease in CD27 expression consistent with progressive differentiation post-vaccination was the only detectable change between the time points studied within individual volunteers (data not shown). Thus, minimal differentiation-associated functional variations would be expected. Interestingly, despite vaccine-mediated expansion of pre-existing memory clonotypes, the observed CD27^+^CD45RO^+^CD57^−^CCR7^−^ phenotype indicates a lack of terminal differentiation and senescence [29]. This is encouraging from the perspective that durable T cell immunity may be feasible using this approach of boosting existing T cell memory with an MVA-vectored vaccine.

MVA-vectored vaccines have the advantage that they can be produced on the large scale required for widespread human vaccination. The low level of polymorphism in NP and M1 across influenza A strains means that a vaccine such as MVA-NP+M1 could provide T cell-mediated protection against all influenza A subtypes.

In summary, we have shown that the novel influenza vaccine candidate MVA-NP+M1 is safe and highly immunogenic in adults over 50 years old. Both CD4^+^ and CD8^+^ memory T cell responses are boosted, and have the capacity to secrete multiple cytokines. Indeed, despite the apparent reduction in immune responsiveness observed in the oldest volunteers in this study, there was still a significant induction of IFN-γ-secreting cells and a significant increase in the proportion of CD4^+^ and CD8^+^ T cells capable of triple cytokine production after vaccination. These enhanced T cell responses could provide heterosubtypic T cell-based immunity against influenza in the elderly.

## Supporting Information

Table S1 **Reagents used for flow cytometry experiments.**
(DOCX)

Table S2 **Statistical comparison of CD4^+^ and CD8^+^ T cell populations quantified according to the number of functions elicited in response to peptides representing the vaccine insert.** P values have not been adjusted for multiple comparisons.
(DOCX)

Protocol S1 **Trial Protocol.**
(PDF)

Checklist S1 **CONSORT Checklist.**
(DOC)
