# Supplementary material for: A T Cell-Inducing Influenza Vaccine for the Elderly: Safety and Immunogenicity of MVA-NP+M1 in Adults Aged over 50 Years
Source: PLoS One. 2012 Oct 31;7(10):e48322. doi: 10.1371/journal.pone.0048322 (PMC3485192; doi:10.1371/journal.pone.0048322)
Supplement: Table S1 — Reagents used for flow cytometry experiments. (DOCX) [file pone.0048322.s001.docx]

**Table S1:** Reagents used for flow cytometry experiments.

| **Panel** | **Supplier** | **Antibody** | **Fluorochrome** | **Clone** |
| --- | --- | --- | --- | --- |
| 1 | eBioscience | αCD3 | Alexa-Fluor-700 | UCHT1 |
| 1 | eBioscience | αCD8 | APC-Alexa-Fluor 780 | RPAT8 |
| 1 | Life Technologies | αCD4 | QD655 | S3.5 |
| 1 | eBioscience | αIFN-γ | FITC | 4S.B3 |
| 1 | eBioscience | αIL-2 | PE | MQ1-17H12 |
| 1 | BD Biosciences | αTNF | PE-Cy7 | MAb11 |
| 1 | eBioscience | αCD107a | PE-Cy5 | eBioH4A3 |
| 1 | Life Technologies | αCD14 | Pacific-Blue | TuK4 |
| 1 | Life Technologies | αCD19 | Pacific-Blue | SJ25-C1 |
| 1 | Life Technologies | Dead cell marker | LIVE/DEAD® Violet |  |
| 2 | eBioscience | αCD3 | Alexa-Fluor-700 | UCHT1 |
| 2 | eBioscience | αCD8 | APC-Alexa-Fluor 780 | RPAT8 |
| 2 | Life Technologies | αCD4 | QD655 | S3.5 |
| 2 | eBioscience | αIFN-γ | PE-Cy7 | 4s.B3 |
| 2 | Life Technologies | IL-10 | FITC | JES3-9D7 |
| 2 | eBioscience | granzyme B | PE | GB11 |
| 2 | eBioscience | IL-17a | PerCP-Cy5.5 | eBio64DEC17 |
| 2 | eBioscience | αCD107a | PE-Cy5 | eBioH4A3 |
| 2 | Life Technologies | αCD14 | Pacific-Blue | TuK4 |
| 2 | Life Technologies | αCD19 | Pacific-Blue | SJ25-C1 |
| 2 | Life Technologies | Dead cell marker | LIVE/DEAD® Violet |  |
| TCR clonotyping | BD Biosciences | αCD3 | APC-H7 | SK7 |
| TCR clonotyping | Life Technologies | αCD4 | PE-Cy5.5 | S3.5 |
| TCR clonotyping | Life Technologies | αCD8 | QD705 | 3B5 |
| TCR clonotyping | Life Technologies | αCD14 | Pacific Blue | TuK4 |
| TCR clonotyping | Life Technologies | αCD19 | Pacific Blue | SJ25-C1 |
| TCR clonotyping | Beckman Coulter | αCD27 | PE-Cy5 | 1A4CD27 |
| TCR clonotyping | Beckman Coulter | αCD45RO | ECD | UCHL1 |
| TCR clonotyping | BD Pharmingen | αCD57 | FITC | NK-1 |
| TCR clonotyping | BD Pharmingen | αCCR7 | PE-Cy7 | 3D12 |
| TCR clonotyping | Life Technologies | Dead cell marker | LIVE/DEAD® Violet |  |
