# Supplementary material for: A T Cell-Inducing Influenza Vaccine for the Elderly: Safety and Immunogenicity of MVA-NP+M1 in Adults Aged over 50 Years
Source: PLoS One. 2012 Oct 31;7(10):e48322. doi: 10.1371/journal.pone.0048322 (PMC3485192; doi:10.1371/journal.pone.0048322)
Supplement: Table S2 — Statistical comparison of CD4+ and CD8+ T cell populations quantified according to the number of functions elicited in response to peptides representing the vaccine insert. P values have not been adjusted for multiple comparisons. (DOCX) [file pone.0048322.s002.docx]

**Table S2:** Statistical comparison of CD4^+^ and CD8^+^ T cell populations quantified according to the number of functions elicited in response to peptides representing the vaccine insert.

| CD4/CD8 | Age group | No. functions | D0 v D7/D21 | P value |
| --- | --- | --- | --- | --- |
| CD4 | 1 (50-59 years) | 4 | 7 | 0·008 |
|  |  | 4 | 21 | 0·0039 |
|  |  | 3 | 7 | 0·0039 |
|  |  | 3 | 21 | 0·0039 |
|  |  | 2 | 7 | 0·0195 |
|  |  | 2 | 21 | 0·0075 |
| CD8 | 1 (50-59 years) | 4 | 7 | 0·0195 |
|  |  | 4 | 21 | 0·0078 |
|  |  | 3 | 7 | 0·0039 |
|  |  | 3 | 21 | 0·038 |
|  |  | 2 | 7 | 0·0078 |
|  |  | 2 | 21 | 0·039 |
| CD4 | 2 (60-69 years) | 3 | 7 | 0·0127 |
|  |  | 3 | 21 | 0·0059 |
|  |  | 2 | 7 | 0·0156 |
|  |  | 2 | 21 | 0·0078 |
| CD4 | 3 (70+ years) | 3 | 7 | 0·0195 |
|  |  | 3 | 21 | 0·0091 |
|  |  | 2 | 7 | 0·0234 |
|  |  | 2 | 21 | 0·0391 |
| CD8 | 3 (70+ years) | 4 | 21 | 0·0223 |
|  |  | 3 | 21 | 0·0078 |
