# Supplementary material for: A T Cell-Inducing Influenza Vaccine for the Elderly: Safety and Immunogenicity of MVA-NP+M1 in Adults Aged over 50 Years
Source: PLoS One. 2012 Oct 31;7(10):e48322. doi: 10.1371/journal.pone.0048322 (PMC3485192; doi:10.1371/journal.pone.0048322)
Supplement: Protocol S1 — Trial Protocol. (PDF) [file pone.0048322.s003.pdf]

UNIVERSITY OF OXFORD

Study Reference: FLU 001

Title: A phase I study to assess the safety and immunogenicity of a new influenza vaccine candidate MVA-NP+M1 in healthy adults.

Sponsor: University of Oxford

Principal Investigator: Prof Adrian V S Hill

Local Safety Monitor: Dr Brian Angus

Version Number: 6.0

07 April 2011

Study Code: Flu001

Eudract Number: 2007-003970-24

Ethics Number: GTAC 143

Principal Investigator:	Professor Adrian V S Hill
Centre:	Centre for Clinical Vaccinology and Tropical Medicine<br>Churchill Hospital, Old Road, Headington<br>Oxford, OX3 7LJ<br>Tel: 01865 857417<br>Fax: 01865 857471<br>Email: adrian.hill@well.ox.ac.uk
Sponsoring Institution	University of Oxford<br>Clinical Trials and Research Governance<br>Manor House, The John Radcliffe Hospital,<br>Headington, Oxford, OX3 9DZ<br>Tel: 01865 743004<br>Fax: 01865 743002<br>Email: heather.house@admin.ox.ac.uk
Clinical Project Manager	Rachel Roberts<br>CCVTM, Oxford<br>Tel: 01865 857418
External Monitor	Appledown Clinical Research Ltd<br>Orchard's End<br>Greenland's Lane<br>Prestwood,<br>Gt. Missenden<br>Bucks.<br>HP16 9QX<br><br>Tel: 01494 867676<br>mobile 07941 271 635

Investigator Agreement

"I have read this protocol and agree to abide by all provisions set forth therein.

I agree to comply with the International Conference on Harmonisation Tripartite Guideline on Good Clinical Practice."

-----------------------------------	-----------------------------------	------------------
Chief Investigator	Investigator Signature	Date
Professor Adrian Hill		

Confidentiality Statement

This document contains confidential information that must not be disclosed to anyone other than the Sponsor, the Investigator Team, and members of the Independent Ethics Committee. This information cannot be used for any purpose other than the evaluation or conduct of the clinical investigation without the prior written consent of Professor Adrian Hill.

TABLE OF CONTENTS

1. REVISION HISTORY 7
2. SYNOPSIS..8
3. ABBREVIATIONS 9
4. BACKGROUND AND RATIONALE 10
4.1 The need for a new vaccine against influenza 10
4.2 The development of a novel vaccination strategy against influenza 10
4.3 The inclusion of NP and M1 in a boosting vaccine..12
4.4 Using recombinant MVA as a boosting vaccine 13
4.5 Recombinant modified vaccinia virus Ankara as a vaccine vector 13
4.6 Clinical studies using recombinant MVA...14
4.7 Study overview 20
4.8 Potential Risks 20
4.9 Known Potential Benefits..21
5. OBJECTIVES..22
5.1 Primary Objectives 22
5.2 Secondary Objectives...22
6. INVESTIGATIONAL PRODUCTS 23
6.1 Vaccine formulation, storage and accountability 23
6.2 MVA-NP+M1 formulation and dose...23
6.3 Vaccine administration...23
6.4 Minimising environmental contamination with Genetically Modified Organisms (GMO) 23
6.5 Vaccine labels 24
7. RECRUITMENT AND WITHDRAWAL OF TRIAL VOLUNTEERS 26
7.1 Volunteers 26
7.2 Informed Consent 26
7.3 Inclusion and Exclusion Criteria...26

7.4 Withdrawal of Volunteers 28
7.5 Compliance with Dosing Regime 29
7.6 Pregnancy 29
8. TRIAL DESIGN 30
8.1 Primary and Secondary Endpoints 30
8.2 Study Groups 30
8.3 Duration of Study 30
8.4 Definition of the Start and End of the Trial 30
9. TREATMENT OF TRIAL VOLUNTEERS 31
9.1 Study procedures 31
9.2 Study visits 32
10. ASSESSMENT OF SCIENTIFIC OBJECTIVES 34
10.1 Primary Outcome Measures 34
10.2 Secondary Outcome Measures 34
11. ASSESSMENT OF SAFETY 35
11.1 Definitions 35
11.2 Causality Assessment 36
11.3 Reporting Procedures for All Adverse Events 37
11.4 Reporting Procedures for Serious Adverse Events 38
11.5 Procedures to be followed in the event of abnormal findings 38
11.6 Local Safety Monitor 39
11.7 Safety Profile Review 39
12. STATISTICS 40
13. QUALITY CONTROL AND QUALITY ASSURANCE PROCEDURES 41
14. ETHICS 42
14.1 Declaration of Helsinki 42
14.2 Good Clinical Practice (GCP) 42
14.3 Informed Consent 42

14.4 Independent Ethics Committee (IEC) ... 42
14.5 Volunteer Confidentiality ... 42
15. DATA HANDLING AND RECORD KEEPING ... 43
15.1 Data Handling ... 43
15.2 Record Keeping ... 43
15.3 Source Data and Case Report Forms (CRFs) ... 43
15.4 Data Protection ... 43
16. FINANCING AND INSURANCE ... 44
16.1 Financing ... 44
16.2 Insurance ... 44
16.3 Compensation ... 44
17. REFERENCES ... 45

1. REVISION HISTORY

Version No.	Date issued	Author(s) of changes
1	17.7.07	Sarah Gilbert
1.1	1.12.07	Sarah Gilbert
1.2	9.1.08	Sarah Gilbert
2.0	15.4.08	Sarah Gilbert
3.0	03.10.08	Matthew Hamill
3.1	23.12.08	Matthew Hamill
4.0	23.10.09	Patrick Lillie
5.0	12.05.10	Patrick Lillie
6.0	07.04.11	Richard Antrobus

2. SYNOPSIS

**Trial Title**	A phase I study to assess the safety and immunogenicity of a new influenza vaccine candidate MVA-NP+M1 in healthy adults.
**Trial Centre**	Centre for Clinical Vaccinology and Tropical Medicine (CCVTM)<br>Old Road, Headington, Oxford, OX3 7LJ, UK
**Trial Identifier**	Flu001
**Clinical Phase**	I
**Trial Design**	Open label observational study
**Trial Population**	Healthy adults aged 18 or over, with no upper age limit
**Planned Sample Size**	Group 1 intradermal<br>12 volunteers total: 1 dose of 5 x 10^7 pfu MVA-NP+M1 day 0<br>Group 2 intramuscular<br>16 volunteers total<br>8 volunteers:1 dose of 5 x 10^7 pfu MVA-NP+M1 day 0<br>8 volunteers:1 dose of 2.5 x 10^8 pfu MVA-NP+M1 day 0<br><br>Group 3 intramuscular upper age range:<br>30 volunteers total, all receiving 1.5 x 10^8 pfu MVA-NP+M1 day 0<br>10 volunteers aged 50-59<br>10 volunteers aged 60-69<br>10 volunteers aged 70 and over
**Follow-up duration**	Approximately 1 year (This is an estimate and may vary in accordance with the specified time windows for each attendance.)
**Planned Trial Period**	2 years (1 year following enrolment of last volunteer)
**Primary Objective**	To assess the safety of a new influenza vaccine, MVA-NP+M1, when administered as a single dose to healthy volunteers.
**Secondary Objective**	To assess the cellular immune response generated by a new influenza vaccine, MVA-NP+M1, when administered as a single dose to healthy volunteers.
**Investigational Products**	MVA-NP+M1
**Form**	Liquid
**Dose**	5 x 10^7 pfu, 1.5x10^8pfu and 2.5 x 10^8 pfu
**Route**	Intradermal (ID) and Intramuscular (IM) injection in the deltoid region of the arm

3. ABBREVIATIONS

**AE**	Adverse event
**CCVTM**	Centre for Clinical Vaccinology and Tropical Medicine
**CRF**	Case Report Form
**ELISPOT**	Enzyme-linked immunospot
**FBC**	Full blood count
**GCP**	Good Clinical Practice
**GMO**	Genetically modified organism
**GTAC**	Gene Therapy Advisory Committee
**HA**	Haemaglutinin
**HBsAg**	Hepatitis B Surface Antigen
**HCG**	Human Chorionic Gonadotrophin
**HCV**	Hepatitis C virus
**HIV**	Human immunodeficiency virus
**HLA**	Human leukocyte antigen
**IDT**	Impfstoffwerk Dessau-Tornau
**IEC**	Independent Ethics Committee
**LSM**	Local safety monitor
**M1**	Matrix protein 1
**MHRA**	Medicines and Healthcare products Regulatory Agency
**MVA**	Modified Vaccinia Virus Ankara
**MVA-NP+M1**	recombinant modified vaccinia virus Ankara expressing influenza nucleoprotein fused to matrix protein 1
**NA**	Neuraminidase
**NHS**	National Health Service
**NP**	Nucleoprotein
**pfu**	Plaque forming units
**SAE**	Serious adverse event
**SOP**	Standard Operating Procedure
**SUSAR**	Suspected unexpected serious adverse reactions

4. BACKGROUND AND RATIONALE

4.1 The need for a new vaccine against influenza

Seasonal influenza has a huge annual impact worldwide, accounting for tens of millions of illnesses, hundreds of thousands of excess hospitalizations, and tens of thousands of excess deaths in the US alone [1]. Recent infections of avian influenza (H5N1) in humans could lead to a new pandemic if the virus acquires the ability to transmit between humans, with potentially devastating effects across the world [2]. Current widely-used vaccines for seasonal influenza A act by stimulating production of antibodies to HA and NA. As these proteins are highly polymorphic, there is very little or no cross-subtype (or heterosubtypic) protection and limited cross-strain protection even within subtypes. Each year a selection of highly prevalent strains are chosen as the basis for vaccine production, for a vaccine that will be used for one season only. This need for constant redesign and remanufacture increases the cost of the vaccines, places limitations on supply [3], and most importantly means that vaccines for newly arising strains can only be produced once the HA and NA sequences of viruses posing the greatest threat to human health have been identified.

Two recent trials of new investigational H5N1 vaccines [4, 5] suggested that a 12 fold greater amount of antigen would be need per vaccine course than with other flu vaccines. This has discouraged further development of vaccines by companies who are concerned that if a pandemic does not occur they will be left with unsold supplies, although other trials have found that the use of adjuvants can reduce the amount of antigen needed [6]. Moreover, the current high rate of diversification of H5N1 strains suggests that vaccines made now may differ so much in their H5 sequence from any pandemic strain that emerges that these vaccines would have little or no efficacy. Avian influenza in humans is currently treated with the anti-viral drug oseltamivir, and this drug is now being stockpiled for use in future pandemics. However, oseltamivir resistant H5N1 virus has now been isolated following human infection [7], so the use of this drug alone may not be sufficient to treat infected individuals and limit the spread of the virus should it become transmissible from human to human.

4.2 The development of a novel vaccination strategy against influenza

Antibodies against the external proteins of influenza can prevent the virus from infecting cells and either prevent infection or limit the spread of infection. However the surface proteins are highly variable and there is little antibody cross-reactivity between variants. Once a cell has been infected with the virus, it is then vulnerable to T cell attack resulting in the destruction of

infected cells so that no more virus can be produced and the infection is controlled. There is evidence from clinical trials of influenza challenge [8], and animal models [9] that T cell responses can protect in the absence of antibodies. Additionally, since T cells can recognise the highly conserved internal proteins of influenza, cross-subtype protection can be achieved [10].

Seasonal influenza infection results in a T cell response to the virus which can protect against subsequent infection. Recent studies in Oxford have shown that 90% of the adult population have detectable T cell responses to one or more influenza antigens (Tao Dong, unpublished). However over the course of a few years these responses decline below protective levels. The new vaccine being tested in this study is designed to boost these T cell responses back to protective levels. Even responses that may be too low to be reliably quantified by currently available assays may still be boosted to high levels by a single dose of recombinant MVA as shown by the tuberculosis vaccine study [11]. Since the internal proteins vary little between influenza subtypes, this could result in a 'universal' vaccine against influenza A. If the need to continually reformulate the vaccine in response to mutations in the viral coat proteins can be removed, the universal vaccine could be produced in large amounts and used more widely than the existing seasonal 'flu vaccines, thus protecting the population against currently circulating viruses and new virus types that are at present only found in avian species.

Figure 1A

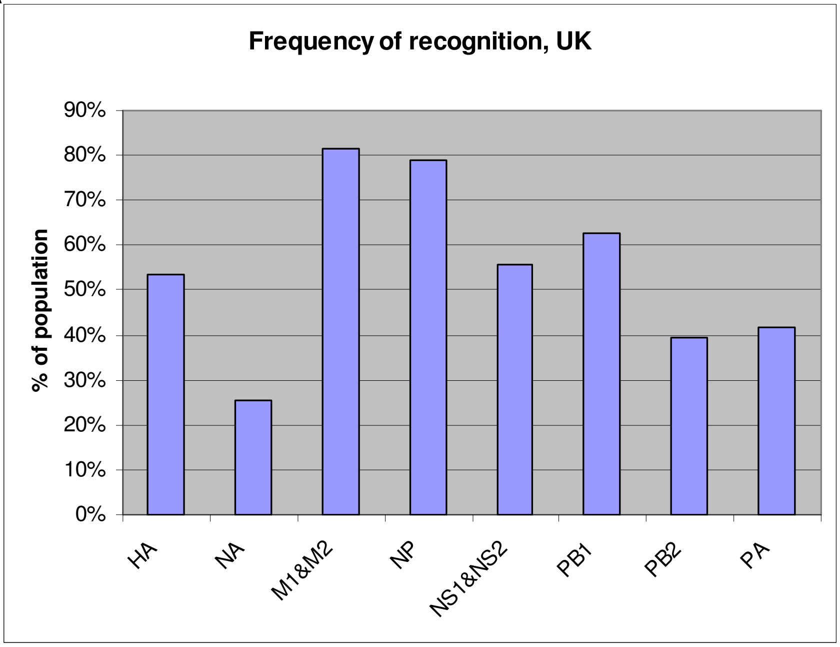

Figure 1B

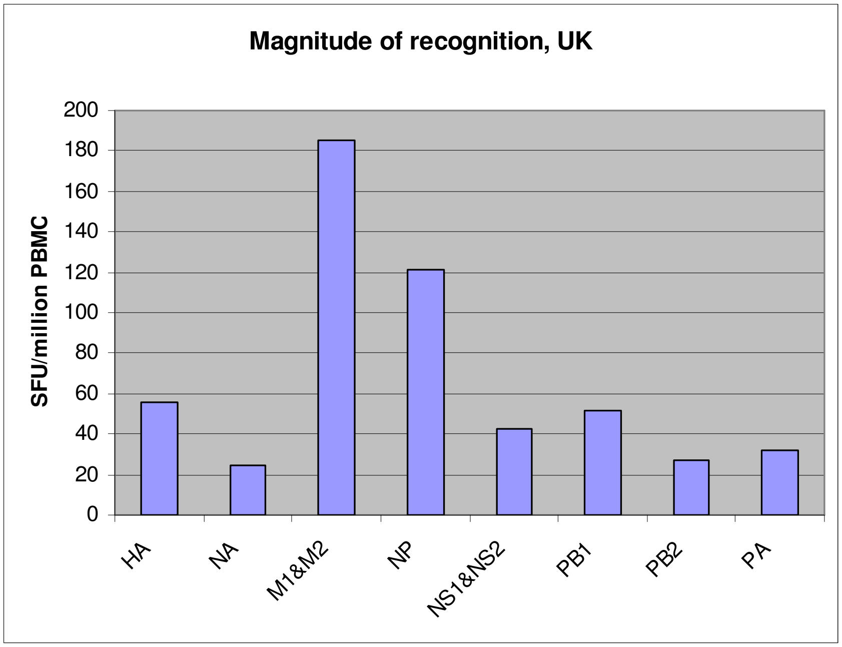

Figure 1. Frequency (A) and Magnitude (B) of T cell responses to influenza antigens in UK adults. Unpublished data supplied by Dr Tao Dong and Miss Laurel Lee, WIMM, Oxford. Responses to M1 and 2 were tested together, but in the small number of volunteers were these were separated the response was predominantly to M1 rather than M2.

**HA**: haemagglutinin	**NA**: neuraminidase	**M1**: matrix protein 1
**M2**: matrix protein 2	**NP**: nucleoprotein	**NS1**: non structural protein 1
**NS2**: non structural protein 2	**PB1**: polymerase subunit B1	**PB2**: polymerase subunit B2
**PA**: polymerase subunit A		

4.3 The inclusion of NP and M1 in a boosting vaccine

There is very little polymorphism of NP and M1 between influenza A isolates. NP is 92% identical between H3N2 and H1N1 strains, and 91% identical between H3N2 and H5N1 strains. M1 is 95% identical between H3N2 and H1N1 strains, and 93% identical between H3N2 and H5N1 strains. This low level of variation appears to allow strong T cell cross-reactivity. For comparison, the sequence of TRAP antigen used in our malaria vaccine differs by 8% from the sequence in the challenge strain, and excellent immunological crossreactivity and cross-strain protection was observed [12]. In addition, each of the antigens (NP and M1) is recognised in a T cell assay by more than 70% of the local population (Tao Dong, unpublished, see Figure 1).

4.4 Using recombinant MVA as a boosting vaccine

Recombinant viral vectors, such as poxviruses and adenoviruses, are a particularly effective way of boosting strong T cell responses to the antigen encoded within them. In our tuberculosis vaccine studies we [11] reported exceptionally high T cell responses in BCG-naïve individuals who were immunised with a single low dose of intradermal MVA expressing Antigen 85A. This secreted antigen is completely conserved between *Mycobacterium tuberculosis* and *M. bovis*, and highly conserved between those species and environmental mycobacteria such as *M. avium*. It is likely that the volunteers had been primed to this antigen by prior exposure to environmental mycobacteria, and that the immunisation with MVA 85A then boosted pre-existing memory responses rather than priming a T cell response *de novo*. Volunteers given BCG followed by MVA85A four weeks later produced even higher T cell responses to Antigen 85A, and these were maintained for longer; at least two years. The same was true of volunteers who had been vaccinated with BCG in the past (median time 18 years prior to MVA vaccination, range 0.5 – 38 years).

In clinical studies with new malaria vaccines, recombinant MVA was found to boost T cell responses in malaria-naïve subjects who had been primed with either a DNA vaccine or recombinant fowlpox expressing the same antigen [12, 13].

4.5 Recombinant modified vaccinia virus Ankara as a vaccine vector

The successful worldwide eradication of smallpox via vaccination with live vaccinia virus highlighted vaccinia as a candidate virus for recombinant use. MVA is a highly attenuated strain of vaccinia virus that is unable to replicate efficiently in human cell lines and most mammalian cells [14]. MVA underwent multiple, fully characterised deletions during more than 570 passages through chicken embryo fibroblast cells [15] including deletions in host range genes and genes encoding cytokine receptors. Viral replication is blocked at a late stage of virion assembly, so, importantly, viral and recombinant protein synthesis is unimpaired [16]. This means that MVA is an efficient single round expression vector, incapable of causing infection in mammals. Replication-deficient recombinant MVA has been seen as an exceptionally safe viral vector. It has been administered to more than 120,000 vaccinees as part of the smallpox eradication programme, with an excellent safety record, despite the deliberate vaccination of high risk groups [15]. This safety in man is consistent with the avirulence of MVA in animal models, where recombinant MVAs have also been shown to be protectively immunogenic as vaccines against viral diseases and cancer. Importantly for a vaccine which may eventually be used in a large proportion of the population, recombinant MVAs expressing HIV antigens have been shown to be safe and

immunogenic in HIV-infected subjects [17-19]. There are now safety data from many recombinant MVAs expressing HIV, malaria and melanoma antigens that are currently in phase I/II trials in both the UK and Africa [17-24].

4.6 Clinical studies using recombinant MVA

MVA-NP+M1 has so far been administered to 43 volunteers; 28 from groups 1, 2a and 2b described in this protocol, FLU001, and 15 in a phase IIa study FLU002. In FLU001, administration of intradermal MVA-NP+M1 resulted in a greater number of local side effects (itching, redness, scaling, pain swelling and warmth at injection site), while the high dose ($2.5x10^8$) intramuscular regime caused a greater number of systemic side effects (malaise, myalgia, rigors). For this reason the $1.5x10^8$ pfu dose administered intramuscularly was chosen for the FLU002 study. The side effect profile of the vaccine in the FLU002 volunteers given 1.5×10^8 pfu has been comparable to other studies with MVA vaccines, with the 15 volunteers experiencing mild systemic symptoms of fever, muscle and joint pains, fatigue and headache, that lasted for a median of 1 day (range 1-6). Local effects consisted of pain, swelling and redness, that lasted for a median of 2 days (range 1-7), and in only 1 volunteer were these symptoms reported as limiting use of the vaccinated limb, lasting for 1 day. In the extension to FLU001 in older volunteers we will therefore continue with the dose of $1.5x10^8$ pfu administered intramuscularly.

MVA85A has been administered (at a dose of 5×10^7 pfu) to 57 healthy volunteers in the UK, 21 healthy adults and 8 healthy infants in The Gambia and 24 adults and 8 adolescents in South Africa, with no vaccine-related serious adverse events (Table 1). In addition to this, MVA85A low dose ($1x10^7$ pfu) and high dose ($1x10^8$ pfu) have each been given intradermally to 12 volunteers with no vaccine-related serious adverse events. All volunteers have temporary local redness with, typically, a 5mm central red area with a paler pink surrounding area that ranges in size from about 1-7mm in diameter and peaks at 48 hours post vaccination. At seven days post vaccination, generally only the central red area remains. This fades over the next few weeks and is not usually apparent at 2 months after vaccination. Febrile symptoms in the first 2 days after vaccination are reported by some volunteers, particularly at higher doses, but are not always accompanied by a measured rise in temperature. A minority of volunteers experienced a mild headache during this period.

MVA85A has also been given to small numbers of adults with latent TB infection or HIV infection. No additional adverse events were reported.

MVA expressing malaria antigens has been used in many studies in the UK and Africa, in adults and children, and these are summarised in Table 2. Adverse events were the same as those reported for MVA-85A; mild or moderate, self-limited local and/or systemic events.

In addition MVA expressing HIV antigens has been administered to 16 chronically HIV-infected adults. Vaccinations were well tolerated and there were no serious adverse events. No breakthrough viraemia occurred after immunisations or throughout follow-up [25]. The same vaccine has been administered to 192 healthy adults [26] and was found to be safe and well-tolerated.

Table 1: Summary of clinical trials with MVA85A (TB antigen)

Country	Volunteer Group	Dose of MVA (pfu)	Volunteers	Status
UK	BCG naïve and Heaf grade 0	$5 \times 10^7 \times 2$ doses	14	Completed
UK	BCG-prime and MVA85A boost (1 month interval)	5×10^7	10	Completed
UK	BCG-prime and MVA85A boost (24 month interval)	5×10^7	5	Completed
UK	BCG-prime and MVA85A boost (10-20 year interval)	5×10^7	12	Completed
UK	BCG-prime and MVA85A dose-optimization	1×10^7<br>1×10^8	12<br>12	Completed
Gambia	BCG naïve and Heaf grade 0	$5 \times 10^7 \times 2$ doses	11	Completed
Gambia	BCG-prime (birth) and MVA85A-boost	5×10^7	10	Completed
S. Africa	BCG naïve or BCG-prime MVA85A-boost	5×10^7	24	Completed
UK	Latent infection	5×10^7	12	Completed
Gambia	BCG vaccinated infants	2.5 and 5×10^7	350	In progress
S. Africa	Healthy BCG-vaccinated adolescents and BCG-naïve or BCG-vaccinated adults	5×10^7	24	Completed
UK	Dose finding	1×10^7 and 1×10^8	24	Completed
UK	Asymptomatic HIV+	5×10^7<br>1×10^8	20	In progress
UK	MVA85A / FP85A Boost	5×10^7	36	In progress

S. Africa	Children and infants	2.5×10^{7}<br>5×10^{7}<br>1×10^{8}	132	In progress
UK	Measurement of Human T-cell Turnover following MVA85A	1×10^{8}	12	In progress
S Africa	MVA85A, in Asymptomatic Volunteers Who Are Infected With Either *Mycobacterium tuberculosis (Mtb.)*, *Human Immunodeficiency Virus (HIV)* or Both	5×10^{7}	36	In progress
Senegal	Asymptomatic HIV+	1×10^{8}	12	In progress

Table 2: Summary of clinical trials with MVA expressing malaria antigens

Country	Volunteer Group	Dose of MVA (pfu)	volunteers	Status
UK	DNA prime (powderject), MVA boost (5 month interval)	$3 \times 10^7 \times 3$ doses	12	Completed
UK	MVA ME TRAP	$3 \times 10^7 \times 3$ doses	6	Completed
UK	DNA prime, MVA boost (3 week interval)	$3 \times 10^7 \times 2$ doses	16	Completed
UK	DNA prime, (powderject), MVA boost (3 week interval)	$3 \times 10^7 \times 2$ doses	9	Completed
Gambia	DNA prime, MVA boost (3 week interval)	$3 \times 10^7 \times 3$ doses	20	Completed
UK	MVA following sporozoite challenge	$5 \times 10^7 \times 2$ doses	4	Completed
UK	Dose ranging	1 or 2.5×10^8	6	Completed
UK	DNA prime, MVA boost (6 week interval)	$15 \times 10^7 \times 2$ doses	7	Completed
Gambia	1-5 year old children dose ranging	6×10^6 or 3×10^7	20	Completed
UK	FP9 ME-TRAP followed by MVA ME-TRAP	1 or 2.5×10^8	12	Completed
Gambia	DNA ME-TRAP, FP9 ME-TRAP and MVA ME-TRAP in adult males	1.5×10^8	29	Completed
UK	Optimisation of prime-boost using DNA, MVA and FP9 based vaccines	5×10^8	17	Completed
UK	Four different combinations of DNA ME-TRAP, MVA ME-TRAP and FP9 ME-TRAP.	1.5×10^8	17	Completed
UK	RTS,S / AS02A and MVA-CSO.	5×10^7 or 1×10^8	24	Completed
Kenya	FP9 ME-TRAP and MVA ME TRAP in semi-immune adult males	2.5×10^8	48	Completed
Gambia	Field Efficacy Study of Heterologous Prime Boost Immunisation in adult men	1.5×10^8	372	Completed
UK	DNA-CS, MVA-CS, DNA-ME TRAP and	1.5×10^8	16	Completed

	MVA-ME TRAP malaria vaccines in healthy adults			
UK	FP9 CSO, FP9 ME-TRAP, MVA CSO, MVA ME-TRAP dose ranging and prime boost	7.5 $x10^7$ ME-TRAP $5x10^7$ CSO	31	Completed
Kenya	Healthy 12-72 month old children. Prime boost FP9-ME TRAP, MVA-ME TRAP	7.5 x 10^7 or 1.5 x 10^8	22	Completed
Kenya	FP9 CS and MVA CS in adult males	5 x 10^7 or 1 x 10^8	30	Completed
Gambia	FP9 CS and MVA CS in adult men	5 x 10^7 or 1 x 10^8	31	Completed
UK	FP9 PP and MVA PP prime boost	1 $x10^8$ or 2 $x10^8$ or 5 $x10^8$	35	Completed
UK	FP9 CS and MVA CS prime boost	5 x 10^8	24	Completed
Gambia	FP9-METRAP and MVA-METRAP in adult males	1 x 10^8	90	Completed
UK	PEV3A and FP9-MVA ME-TRAP	1.5 x 10^8	27	Completed
Kenya	FP9:ME-TRAP and MVA:ME-TRAP in children aged 1-6 years	1.5 x 10^8 or 7.5 x 10^7	346	Completed
UK	MVA ME-TRAP and AdCh63 ME-TRAP	2 x 10^8	16	In progress

Table 3: Summary of clinical trials with MVA expressing Influenza antigens

**Country**	**Volunteer Group**	**Dose of MVA (pfu)**	**volunteers**	**Status**
UK	MVA-NP+M1	ID 5 x 10^7	12	In progress
		IM 5 x 10^7 and 2.5 $x10^8$	16	
UK	MVA-NP+M1	IM 1.5 x 10^8	15	In progress

4.7 Study overview

This is an open label phase I study, to assess the safety of a novel influenza vaccine, MVA-NP+M1. All volunteers recruited will be healthy. Twelve volunteers will be administered with a single dose of 5×10^7 pfu of MVA-NP+M1 via the ID route (group 1). Sixteen volunteers will receive IM MVA-NP+M1. The first 8 volunteers will be administered a single dose of 5×10^7 pfu of MVA-NP+M1 (group 2) followed by a further eight receiving 2.5×10^8 pfu of MVA-NP+M1 (group 3). A further group of 30 volunteers, stratified by age (10 volunteers 50-59, 10 60-69, 1070 and above) will be administered with a single IM dose of 1.5×10^8 pfu of MVA-NP+M1. Safety data will be collected. The secondary aim of this study will be to assess the cellular immune responses generated by each dose.

4.8 Potential Risks

The general risks to participants in this Phase I study are associated with phlebotomy and with vaccination. The volume of blood drawn over the 1-year study period (up to 650mL) should not compromise these otherwise healthy volunteers. Potential risks include local and systemic reactions, which are described below. As vaccine-related side effects are believed to be related more to the vector used than the specific insert, it is expected that MVA-NP+M1 will have a similar side effect profile to recombinant MVA viruses encoding other antigens. To date the safety data of all 43 volunteers to receive MVA-NP+M1 has been excellent, with no serious adverse events recorded.

Local reactions

Mild tenderness, bruising, light-headedness or, rarely, syncope, may result from venepuncture. Vaccination usually precipitates a local inflammatory reaction. This may include redness, swelling, scaling, tenderness, or itching. In previous studies using recombinant MVA vaccines, these local reactions have spontaneously resolved within weeks.

Systemic Reactions

Systemic reactions that could potentially occur following immunisation with a recombinant MVA vaccine include a flu-like illness with low-grade fever, chills and malaise. In general, it appears that the frequency of systemic side effects in response to recombinant MVA vaccines is affected by a preceding poxvirus vaccination, with the proportion of volunteers experiencing any systemic side effects after the first vaccination being 69%, decreasing to 37% after the second and 22% after a third immunisation. As with any other vaccine, the

Guillain-Barré syndrome, (GBS) or immune mediated reactions that can lead to organ damage may occur. However, this has never been seen with a recombinant MVA vaccine. As with any vaccine, serious allergic reactions may occur.

4.9 Known Potential Benefits

Volunteers will not benefit directly from participation in this study. However, it is hoped that the information gained from this study will contribute to the development of a safe and effective influenza vaccine regime. The only benefits for participants would be information about their general health status.

5. OBJECTIVES

5.1 Primary Objectives

To assess the safety of a new influenza vaccine, MVA-NP+M1, when administered to healthy volunteers.

5.2 Secondary Objectives

To assess the cellular immune response generated by MVA-NP+M1, when administered to healthy volunteers.

6. INVESTIGATIONAL PRODUCTS

The vaccine to be used in this study is MVA-NP+M1.

6.1 Vaccine formulation, storage and accountability

MVA-NP+M1 is manufactured under Good Manufacturing Practice conditions by Impfstoffwerk Dessau-Tornau (IDT), Germany. The vaccine is supplied as liquid in glass vials for intradermal and intramuscular administration and are stored, between –70^{0}C and –90^{0}C, in a locked freezer, at the University of Oxford, Churchill Hospital. All movements of the study vaccines between IDT and the University of Oxford and between the locked freezer and clinic room will be documented.

6.2 MVA-NP+M1 formulation and dose

Each vial of MVA-NP+M1 contains 600 microlitres volume at a concentration of 1.3 x 10^8 pfu/ml in 10mM Tris buffer. The doses of MVA-NP+M1 to be used in this study will be 5 x10^7 pfu, 1.5 x10^8 pfu and 2.5 x 10 ^{8} pfu.

6.3 Vaccine administration

On vaccination day, vaccine will be allowed to thaw to room temperature and administered within 1 hour. The vaccine will be administered either intradermally or intramuscularly into the deltoid region of the arm. The investigators will wear gloves and eye protection. Volunteers will stay in the unit for 30 minutes (±10 minutes) after vaccination. During administration of the vaccine, medicines and resuscitation equipment will be immediately available for the management of anaphylaxis.

As volunteers may already have T cell responses to NP and M1, these will be measured by elispot assay on blood taken at both the screening visit and on the day of vaccination, in order to determine the existing responses, and subsequently the increase in T cell responses induced by vaccination.

6.4 Minimising environmental contamination with Genetically Modified Organisms (GMO)

In order to minimise dissemination of the recombinant vectored vaccine virus into the environment, the inoculation site will be covered with a dressing after immunisation. This should absorb any virus that may leak out through the needle track. The dressing will be removed from the injection site after 30 minutes (± 10 minutes) and will be disposed as GMO

waste by autoclaving, in accordance with the relevant Standard Operating Procedure (SOP) and current standard UK practice.

6.5 Vaccine labels

The vaccines will be labelled as detailed below:

Box label

**FOR CLINICAL TRIAL USE ONLY**<br>Trial number Flu 001<br>Prof. Adrian Hill, CCVTM, University of Oxford, Churchill Hospital, Oxford, OX3 7LJ, Ph: 01865 857401<br>**MVA-NP+M1 VACCINE 0.6 ml**<br>(containing 1.3 x 10^8 pfu/ml)<br>For Intramuscular Injection<br>Batch no. 01 09 07 Expiry date: 24.09.10<br>STORE AT <-70ºC	**FOR CLINICAL TRIAL USE ONLY**<br>Trial number Flu 001<br>Prof. Adrian Hill, CCVTM, University of Oxford, Churchill Hospital, Oxford, OX3 7LJ, Ph: 01865 857401<br>**MVA-NP+M1 VACCINE 0.6 ml**<br>(containing 1.3 x 10^8 pfu/ml)<br>For Intradermal Injection<br>Batch no. 01 09 07 Expiry date: 24.09.10<br>STORE AT <-70ºC

Vial label

CLINICAL TRIAL: Flu001<br>MVA NP+M1 VACCINE 0.6ml<br>Vial contains 1.3 x 10^8 pfu/ml<br>Vial No: *** For Intradermal Injection<br>Batch no: 010907 Store at –80oC<br>Volunteer no: _____ Expiry Date: 24.09.10		**FOR CLINICAL TRIAL USE ONLY**<br>**Chief Investigator: Prof Adrian Hill**<br>**University of Oxford**<br>**CCVTM, Old Road, Oxford. OX3 7LJ**<br>**Tel: 01865 617610**<br>**Fax: 01865 857471**

CLINICAL TRIAL: Flu001

MVA NP+M1 VACCINE 0.6ml

Vial contains 1.3×10^{8} pfu/ml

Vial No: *** For Intramuscular Injection

Batch no: 010907 Store at –80°C

Volunteer no: _____ Expiry Date: 24.09.10

FOR CLINICAL TRIAL USE ONLY

Chief Investigator: Prof Adrian Hill

University of Oxford

CCVTM, Old Road, Oxford. OX3 7LJ

Tel: 01865 617610

Fax: 01865 857471

7. RECRUITMENT AND WITHDRAWAL OF TRIAL VOLUNTEERS

7.1 Volunteers

All subjects in this study will be recruited through a combination of poster campaigns, website advertising, email, mail-shots, radio announcements, newspaper adverts and flyers distributed from stalls or stands at exhibitions or fairs.

7.2 Informed Consent

All volunteers will sign and date the informed consent form before any study specific procedures are performed. The information sheet will be made available to the volunteer at least 24 hours prior to the screening visit. At the screening visit, the volunteer will be fully informed of all aspects of the trial, the potential risks and their obligations. The following general principles will be emphasised:

- Participation in the study is entirely voluntary
- Refusal to participate involves no penalty or loss of medical benefits
- The volunteer may withdraw from the study at any time
- The volunteer is free to ask questions at any time to allow him or her to understand the purpose of the study and the procedures involved
- The study involves research of an investigational vaccine
- There is no direct benefit for participating
- The volunteer's GP will be contacted to corroborate their medical history

The aims of the study and all tests to be carried out will be explained. The volunteer will be given the opportunity to ask about details of the trial, and will then have time to consider whether or not to participate. If they do decide to participate, they will sign and date two copies of the consent form, one for them to take away and keep, and one to be stored in the Case Report Form (CRF). These forms will also be signed and dated by the one of the Investigators.

7.3 Inclusion and Exclusion Criteria

This study will be conducted in healthy adults, who meet the following inclusion and exclusion criteria.

Inclusion Criteria

The volunteer must satisfy all the following criteria to be eligible for the study:

- Healthy adult 18 or over, no upper age limit
- Resident in or near Oxford for the duration of the vaccination study
- Able and willing (in the Investigators' opinions) to comply with all study requirements
- Willing to allow the investigators to discuss the volunteer's medical history with their General Practitioner
- For females, a negative pregnancy test on the day of vaccination and agreement to practice effective contraception for the duration of the study, or greater than 12 months since last menstruation, in the absence of hormonal contraception (i.e. post menopausal).
- Agreement to refrain from blood donation during the course of the study
- Written informed consent

Exclusion Criteria

The volunteer may not enter the study if any of the following apply:

- Participation in another research study involving an investigational product in the 30 days preceding enrolment, or planned use during the study period
- Prior receipt of a recombinant MVA vaccine
- Administration of immunoglobulins and/or any blood products within the three months preceding the planned administration of the vaccine candidate
- Any confirmed or suspected immunosuppressive or immunodeficient state, including HIV infection; asplenia; recurrent, severe infections and chronic (more than 14 days) immunosuppressant medication within the past 6 months (inhaled and topical steroids are allowed)
- History of allergic disease or reactions likely to be exacerbated by any component of the vaccine, e.g. egg products
- Any history of anaphylaxis in reaction to vaccination
- History of cancer (except basal cell carcinoma of the skin and cervical carcinoma in situ)
- History of serious psychiatric condition
- Any chronic illness requiring ongoing or awaiting hospital specialist supervision, other than minor surgical procedures and follow up of surgery over 6 months prior to screening.

- Suspected or known current injecting drug or alcohol abuse (as defined by an alcohol intake of greater than 42 units every week)
- Seropositive for hepatitis B surface antigen (HBsAg)
- Seropositive for hepatitis C virus (antibodies to HCV)
- For females, pregnancy, lactation or willingness/intention to become pregnant during the study
- Any other significant disease, disorder or finding, which, in the opinion of the Investigators, may either put the volunteer at risk because of participation in the study, or may influence the result of the study, or the volunteer's ability to participate in the study.
- Any clinically significant abnormal finding on screening biochemistry or haematology blood tests or urinalysis, as determined by the investigators.

7.4 Withdrawal of Volunteers

Volunteers may withdraw or be withdrawn for any of the reasons given below. The reason for withdrawal will be recorded in the CRF. If withdrawal is due to an adverse event, appropriate follow-up visits or medical care will be arranged until the adverse event has resolved or stabilised. Any volunteer who is withdrawn from the study may be replaced, if that is possible within the specified time frame. The Local Safety Monitor (LSM) may recommend withdrawal of volunteers (see Section 11.6).

Discontinuation Criteria

In accordance with the current revision of the Declaration of Helsinki (amended October 2000, with additional footnotes added 2002 and 2004) and any other applicable regulations, a volunteer has the right to withdraw from the study at any time and for any reason and is not obliged to give his or her reasons for doing so. The Investigators may withdraw the volunteer at any time in the interests of the volunteer's health and well-being. In addition the volunteer may withdraw/be withdrawn for any of the following reasons:

- Administrative decision by the Investigators
- Pregnancy
- Ineligibility (either arising during the study or retrospective, having been overlooked at screening)
- Significant protocol deviation
- Volunteer non-compliance with treatment regime or study requirements

- An adverse event which requires discontinuation of the vaccination regimen or results in inability to continue to comply with study procedures (see below)

Contraindications to vaccination

The following adverse events constitute contraindications to administration of vaccine at that point in time. If any one of these adverse events occurs at the time scheduled for vaccination, the volunteer may be vaccinated at a later date, or withdrawn at the discretion of the Investigators:

- Acute disease at the time of vaccination, defined as the presence of a moderate or severe illness with or without fever
- Temperature of ≥37.5℃ at the time of vaccination

(All vaccines can be administered to persons with a minor illness, such as diarrhoea or mild upper respiratory infection, with or without low-grade febrile illness, i.e., temperature of <37.5℃)

7.5 Compliance with Dosing Regime

All doses in this vaccine study will be administered by the one of the Investigators and recorded in the CRF. The study medication will be at no time in the possession of the volunteer and compliance will not, therefore, be an issue.

7.6 Pregnancy

Should a volunteer become pregnant during the trial, she will be followed according to the protocol, but in addition will be followed until pregnancy outcome.

8. TRIAL DESIGN

8.1 Primary and Secondary Endpoints

The specific endpoints for safety and reactogenicity will be actively and passively collected data on adverse events. The specific endpoints for immunogenicity will be markers of cell-mediated immunity.

8.2 Study Groups

This is an open Phase 1 study of a recombinant MVA expressing NP and M1 in three groups of healthy volunteers (12 in the ID arm, 16 in the IM arm and 30 in the age stratified IM arm). The first IM arm will be split into 2 groups of 8. The first 8 will receive the low dose vaccine, followed by the second 8 who will receive the high dose vaccine. The 3rd group will be split into 3 groups of 10 volunteers in the age ranges 50-59, 60-69 and 70 and above.

8.3 Duration of Study

Each volunteer will attend for one vaccination, on the day of enrolment. The follow up visits are as described in section 9.2, with a final visit approximately one year after enrolment.

8.4 Definition of the Start and End of the Trial

The start of the trial is defined as the date of the first vaccination of the first volunteer. The end of the trial is the date of the last visit of the last volunteer.

9. TREATMENT OF TRIAL VOLUNTEERS

9.1 Study procedures

Procedures will be performed on the visit time points indicated in the schedule of procedures. Additional procedures or laboratory tests may be performed, at the discretion of the Investigators e.g. urine microscopy in the event of positive urinalysis.

Observations

Observations which will be documented are pulse, blood pressure and temperature.

Blood Tests

Blood will be drawn for the following laboratory tests:

1. At the Oxford Radcliffe Hospitals NHS Trust Laboratories, using NHS standard procedures:

- **Haematology;** Full Blood Count
- **Biochemistry;** Sodium, Potassium, Urea, Creatinine, Albumin, Liver Function Tests, Glucose
- **Diagnostic serology;** HBsAg, HCV antibodies, HIV antibodies (Counselling will be given prior to testing blood for these blood-borne viruses)
- **Immunology;** Human Leucocyte Antigen (HLA) typing

2. At University of Oxford research laboratories:

Exploratory Immunology; Ex vivo Elispot assays for interferon gamma will be performed. Other exploratory immunological assays, including Elispot assays for interleukin-2 and tumour necrosis factor alpha, may be performed at the discretion of the Investigators. These may include gene expression studies. Some of this blood will be required for immediate use and the remainder stored for up to 15 years as a future research source, with the consent of the volunteers.

Urinalysis

Urine will be tested for protein and glucose at screening. For pre-menopausal female volunteers only, urine will be tested for beta-Human Chorionic Gonadotrophin (HCG) at screening and immediately prior to vaccination.

Vaccinations

Before each vaccination, the on-going eligibility of the volunteer will be reviewed. The vaccine will be administered as described above in section 6.4. The injection site will be covered with a sterile dressing and the volunteer will stay in the CCVTM for 30 minutes (± 10 minutes) after vaccination, in case of immediate adverse events. Observations will be taken 30 minutes (± 10 minutes) after vaccination and the sterile dressing removed and injection site inspected. An oral thermometer, tape measure and diary card will be given to each volunteer, with instructions on use.

9.2 Study visits

The study visits and procedures will be undertaken by one of the Investigators. The procedures to be included in each visit are documented in the schedules of attendances (tables 3 and 4). Each visit is assigned a time point and a window period, within which the visit will be conducted. These can be found in the schedules of attendances.

Screening Visit

All potential volunteers will have a screening visit, which may take place up to 90 days prior to vaccination. Informed consent will be taken before screening, as described in section 7.2. If consent is obtained, the screening procedures indicated in the schedule of procedures will be undertaken.

Visits for volunteers

Day 0 Enrolment and first vaccination

The eligibility of the volunteer will be reviewed at the end of the screening visit and again when all results from the screening visit have been considered. If eligible, a day 0 visit will be scheduled for the volunteer to receive the vaccine. Volunteers will not be considered enrolled in the study until they have received their vaccine. The vaccine will be administered in the non-dominant arm or both arms for high dose IM vaccination.

Subsequent visits (day 2 and weeks 1, 3, 8, 12, 24, 52)

On subsequent visits, the volunteers will be assessed for local and systemic adverse events, using a diary card, interim history, physical examination and blood tests at the time points indicated in the schedule of attendances. Blood will also be taken for exploratory immunology analysis.

Table 3: Schedule of Attendances

	S	V							
**Attendance number**	1	2	3	4	5	6	7	8	9
**Timeline (weeks+days)**		0	0+2	1	3	8	12	24	52
**Window (days)**			±1	±2	±7	±7	±7	±14	±28
**Inclusion / Exclusion criteria**	X								
**Informed consent**	X								
**Medical History**	X	(x)	(x)	(x)	(x)	(x)	(x)	(x)	(x)
**Physical Examination**	X	(x)	(x)	(x)	(x)	(x)	(x)	(x)	(x)
**Urinalysis**	X								
**β-HCG urine test**	X	X							
**Review contraindications**	X	X							
**Vaccination**		X							
**Observations**	X	X	X	X	X	X	X	X	X
**Local & systemic events / reactions**		X	X	X	X	X	X	X	X
**Diary cards provided**		X							
**Diary cards collected**				X					
**HLA typing (mL)**		4							
**HBV,HCV,HIV (mL)**	5								
**Haematology (mL)**	2			2			2		
**Biochemistry (mL)**	4			4			4		
**Glucose (mL)**	4			4			4		
**Exploratory immunology**	60	60	20	60	60	60	60	60	60
**Blood volume per visit (mL)**	75	64	20	70	60	60	70	60	60
**Cumulative blood volume (mL)**	75	139	159	229	289	349	419	479	539

S screening visit

V vaccination visit

(x) If considered necessary, emphasising any acute complaints

10. ASSESSMENT OF SCIENTIFIC OBJECTIVES

10.1 Primary Outcome Measures

To assess the safety of a candidate influenza vaccine, MVA-NP+M1 in healthy adult volunteers. The specific endpoints for safety and reactogenicity will be actively and passively collected data on adverse events.

10.2 Secondary Outcome Measures

To assess the immunogenicity of a candidate influenza vaccine, MVA-NP+M1 in healthy adult volunteers.

11. ASSESSMENT OF SAFETY

Safety will be assessed by the frequency, incidence and nature of adverse events and serious adverse events arising during the study.

11.1 Definitions

Adverse Event (AE)

An AE is any untoward medical occurrence in a volunteer, including a dosing error, which may occur during or after study vaccination and does not necessarily have to have a causal relationship with vaccination. An AE can therefore be any unfavourable and unintended sign (including an abnormal laboratory finding), symptom or disease temporally associated with study vaccination, whether or not considered related to study vaccination.

Adverse Drug Reaction (ADR)

An ADR is any untoward or unintended response to a medicinal product. This means that a causal relationship between the study medication and an AE is at least a reasonable possibility, i.e., the relationship cannot be ruled out.

Unexpected Adverse Reaction

An unexpected adverse reaction is where the nature or severity is not consistent with the Investigator's Brochure.

Serious Adverse Event (SAE) or Adverse Drug Reaction

An SAE is an AE that results in any of the following outcomes, whether or not considered related to the vaccine.

- Death (i.e., results in death from any cause at any time)
- Life-threatening event (i.e., the volunteer was, in the view of the Investigators, at immediate risk of death from the event that occurred). This does not include an AE that, if it occurred in a more serious form, might have caused death.
- Persistent or significant disability or incapacity (i.e. substantial disruption of one's ability to carry out normal life functions).
- Hospitalisation, regardless of length of stay, even if it is a precautionary measure for continued observation. Hospitalisation (including inpatient or outpatient hospitalization for an elective procedure) for a pre-existing condition that has not worsened unexpectedly does not constitute a serious AE.

- An important medical event (that may not cause death, be life threatening, or require hospitalization) that may, based upon appropriate medical judgment, jeopardize the volunteer and/or require medical or surgical intervention to prevent one of the outcomes listed above. Examples of such medical events include allergic reaction requiring intensive treatment in an emergency room or clinic, blood dyscrasias, or convulsions that do not result in inpatient hospitalization.
- Congenital anomaly or birth defect.

Suspected Unexpected Serious Adverse Reactions (SUSARs)

A SUSAR is different from an SAE in that it is unexpected and thought to be related to the investigational product. Reports of any SUSAR will be sent to the MHRA and GTAC within 7 days for fatal and life-threatening cases and within 15 days for all other SUSARs. Administration of further vaccines within the trial will be suspended until a safety review is convened.

Expected Adverse Drug Reactions

Expected local reactions to the vaccine will not be recorded as AEs, but will be recorded in the CRFs. These include local redness, swelling, tenderness, or itching.

Expected Serious Adverse Events

No serious adverse events are expected in this study. To date there have been no expected serious adverse events from the current intramuscular or intradermal MVA-NP+M1 vaccine.

11.2 Causality Assessment

For every AE, an assessment of the relationship of the event to the administration of the vaccine will be undertaken. An intervention-related AE refers to an AE for which there is a probable or definite relationship to administration of a vaccine. An interpretation of the causal relationship of the intervention to the AE in question will be made, based on the type of event; the relationship of the event to the time of vaccine administration; and the known biology of the vaccine therapy (table 5).

Table 5: Guidelines for assessing the relationship of vaccine administration to an AE

1	**No Relationship**	No temporal relationship to study product ***and***<br>Alternate etiology (clinical state, environmental or other interventions); ***and***<br>Does not follow known pattern of response to study product
2	**Possible**	Reasonable temporal relationship to study product; ***or***<br>Event not readily produced by clinical state, environmental or other interventions; ***or***<br>Similar pattern of response to that seen with other vaccines
3	**Probable**	Reasonable temporal relationship to study product; ***and***<br>Event not readily produced by clinical state, environment, or other interventions ***or***<br>Known pattern of response seen with other vaccines
4	**Definite**	Reasonable temporal relationship to study product; ***and***<br>Event not readily produced by clinical state, environment, or other interventions; ***and***<br>Known pattern of response seen with other vaccines

11.3 Reporting Procedures for All Adverse Events

All AEs occurring up to the week 12 post vaccination visit, observed by the Investigators or reported by the patient, whether or not attributed to study medication, will be reported in the CRF. After the week 12 visit (attendance 7) only data on SAE's and SUSARS will be entered into the CRF. All AEs that result in a patient's withdrawal from the study will be followed up until a satisfactory resolution occurs, or until a non-study related causality is assigned. All deaths occurring during the study will be reported to the Sponsor. For all deaths, available autopsy reports and relevant medical reports will be made available for reporting to the relevant authorities. The severity of events will be assessed according to the scales in table 6.

Table 6: Scale for assessing the severity of AEs

Scale	Description	Definition
**0**		Absence of the indicated symptom
**1**	Mild	Awareness of a symptom but the symptom is easily tolerated
**2**	Moderate	Discomfort enough to cause interference with usual activity
**3**	Severe	Incapacitating; unable to perform usual activities; requires absenteeism or bed rest
**4**	Serious	Life-threatening

11.4 **Reporting Procedures for Serious Adverse Events**

In order to comply with current regulations on serious adverse event reporting to Health Authorities, the event will be documented accurately and notification deadlines respected. SAEs will be reported to an internal safety group, within 1 working day of the Investigators being aware of their occurrence, as described in internal SOP-TC009. SAEs will not normally be reported to GTAC, unless there is a clinically important increase in occurrence rate, an unexpected outcome, or a new event that is likely to affect safety of trial volunteers. In addition to the expedited reporting above, the Investigators shall submit once a year throughout the study, or on request, a safety report to the Competent Authority and Ethics Committee.

11.5 **Procedures to be followed in the event of abnormal findings**

Abnormal clinical findings from medical history, examination or blood tests, will be assessed as to their clinical significance. If a test is deemed clinically significant, it may be repeated, to ensure it is not a single occurrence. If a test remains clinically significant, the volunteer will be informed and appropriate medical care arranged as appropriate and with the permission of the volunteer. Decisions to exclude the volunteer from the enrolling in the trial or to withdraw a volunteer from the trial will be at the discretion of the Investigators, following procedures for adverse events as described in section 11.1.

11.6 Local Safety Monitor

A Local Safety Monitor (LSM) will be appointed to provide real-time safety oversight. The LSM will review SAEs if deemed possibly, probably or definitely related to vaccination. The LSM will be notified within 1 working day of the Investigators' being aware of their occurrence. The LSM has the power to terminate the study if deemed necessary following a vaccine-related SAE.

11.7 Safety Profile Review

The safety profile will be assessed on an ongoing basis by the Investigators and specifically after group one has been vaccinated, before enrolling volunteers into group two. An internal safety group will review safety issues and SAEs as they arise. An internal group was deemed appropriate due to extensive experience of this group with this viral vector and the expected adverse events.

12. STATISTICS

This is primarily a safety study. A secondary outcome is to determine substantial differences in the magnitude of immune responses before and after vaccination.

Determination of the Sample Size

This is an observational and descriptive safety study, where 12 subjects will be initially vaccinated intradermally with low dose MVA-NP+M1 followed by 16 subjects intramuscularly. The IM volunteers will be split into 2 equal sized groups, with 8 per group. Group 1 will be vaccinated with $5x10^7$ pfu MVANP+M1 and group 2 will be vaccinated with $2.5x10^8$ pfu MVANP+M1. A further 30 volunteers over the age of 50 will be vaccinated with 1.5×10^8 pfu MVA-NP+_M1, with 10 volunteers in each of 3 aged defined groups (50-59, 60-69 and 70 and above). This sample size should allow determination of the magnitude of the outcome measures, especially of serious and severe adverse events, rather than aiming to obtain statistical significance.

12.1 Statistical Methods

The analysis will be only descriptive, as the sample size does not allow to perform comparison.

13. QUALITY CONTROL AND QUALITY ASSURANCE PROCEDURES

Data will be evaluated for compliance with the protocol and accuracy in relation to source documents. The study will be conducted in accordance with procedures identified in the protocol. SOPs will be used at the clinical and laboratory site. Regular monitoring will be performed according to the requirements of the Medicines for Human Use (Clinical Trial) Regulations 2004 and ICH Good Clinical Practice (GCP). Following written standard operating procedures, the monitors will verify that the clinical trial is conducted and data are generated, documented and reported in compliance with the protocol, GCP and the applicable regulatory requirements. The Investigator site will provide direct access to all trial related source data/documents and reports for the purpose of monitoring and auditing by the sponsor, and inspection by local and regulatory authorities.

14. ETHICS

14.1 Declaration of Helsinki

The Investigators will ensure that this study is conducted in full conformity with the current revision of the Declaration of Helsinki (last amended October 2000, with additional footnotes added 2002 and 2004).

14.2 Good Clinical Practice (GCP)

The Investigators will ensure that this study is conducted in accordance with the ethical principles that have their origin in the Declaration of Helsinki, and that are consistent with ICH GCP and the requirements of the Medicines for Human Use (Clinical Trial) Regulations 2004.

14.3 Informed Consent

Written, informed consent will be obtained, as described in section 7.2.

14.4 Independent Ethics Committee (IEC)

A copy of the protocol, proposed informed consent form, other written volunteer information and the proposed advertising material will be submitted to an IEC (GTAC) for written approval. The Investigators will submit and, where necessary, obtain approval from the GTAC for all subsequent substantial amendments to the protocol and informed consent document. The Investigators will notify deviations from the protocol or SAEs occurring at the site to the sponsor and will notify the GTAC of these in accordance with local procedures.

14.5 Volunteer Confidentiality

The Investigators will ensure that the volunteer’s anonymity is maintained. All documents will be stored securely and kept in strict confidence in compliance with the Data Protection Act. All computer entry and networking programs will be done with coded numbers and initials only. Only the sponsor representative, Investigators, the clinical monitor, the GTAC and the MHRA will have access to the records. Photographs taken of vaccination sites (with the volunteer’s written, informed consent) will not include the volunteer’s face and will be identified by the volunteer’s 3-digit identification number only. Once developed, photographs will be stored as confidential records, as above. This material may be shown to other professional staff, used for educational purposes, or included in a scientific publication.

15. DATA HANDLING AND RECORD KEEPING

15.1 Data Handling

The Principal Investigator will have overall responsibility for receiving, entering, cleaning, querying, analysing and storing all data that accrues from the study, but these tasks may be delegated to other Investigators. The data will be entered into the volunteers' CRFs. This includes safety data, laboratory data (both clinical and immunological) and outcome data.

15.2 Record Keeping

The Investigators will maintain appropriate medical and research records for this trial, in compliance with the requirements of the Medicines for Human Use (Clinical Trial) Regulations 2004, ICH E6 GCP and regulatory and institutional requirements for the protection of confidentiality of volunteers. The principal Investigator, co-investigators and clinical research nurses will have access to records. The Investigators will permit authorized representatives of the sponsor(s), and regulatory agencies to examine (and when required by applicable law, to copy) clinical records for the purposes of quality assurance reviews, audits and evaluation of the study safety and progress.

15.3 Source Data and Case Report Forms (CRFs)

All protocol required information will be collected in CRFs designed by the Investigators. All source documents will be filed in the CRF. Source documents are original documents, data, and records from which the volunteer's CRF data are obtained. For this study, these will include, but are not limited to, volunteer consent form, blood results, GP response letters, laboratory records, diaries, and correspondence. In the majority of cases, CRF entries will be considered source data as the CRF is the site of the original recording (i.e., there is no other written or electronic record of data). In this study this will include, but is not limited to medical history, medication records, vital signs, physical examination records, urine assessments, blood results, adverse event data and details of vaccinations. All source data and volunteer CRFs will be stored securely.

15.4 Data Protection

The study protocol, documentation, data and all other information generated will be held in strict confidence. No information concerning the study or the data will be released to any unauthorized third party, without prior written approval of the sponsor.

16. FINANCING AND INSURANCE

16.1 Financing

The study will be funded by a research grant from the Wellcome Trust held by Dr Sarah Gilbert.

16.2 Insurance

Indemnity and/or compensation for harm arising specifically from an accidental injury, and occurring as a consequence of the Research Subject's participation in the Trial for which the University of Oxford is the Research Sponsor will be covered by the University of Oxford.

16.3 Compensation

Volunteers will be compensated for their time and for the inconvenience caused by procedures. Volunteers will be compensated £320 (calculated as £12 travel allowance per visit; £8 per hour; £10 per blood donation plus a £30 trial completion bonus).

17. REFERENCES

1. Molinari, N.A., et al., *The annual impact of seasonal influenza in the US: measuring disease burden and costs.* Vaccine, 2007. **25**(27): p. 5086-96.
2. Babakir-Mina, M., et al., *Influenza virus A (H5N1): a pandemic risk?* New Microbiol, 2007. **30**(2): p. 65-78.
3. Bardenheier, B.H., et al., *Influenza vaccine supply, 2005-2006: did we come up short?* BMC Health Serv Res, 2007. **7**: p. 66.
4. Bresson, J.L., et al., *Safety and immunogenicity of an inactivated split-virion influenza A/Vietnam/1194/2004 (H5N1) vaccine: phase I randomised trial.* Lancet, 2006. **367**(9523): p. 1657-64.
5. Treanor, J.J., et al., *Safety and immunogenicity of an inactivated subvirion influenza A (H5N1) vaccine.* N Engl J Med, 2006. **354**(13): p. 1343-51.
6. Stephenson, I., et al., *Cross-reactivity to highly pathogenic avian influenza H5N1 viruses after vaccination with nonadjuvanted and MF59-adjuvanted influenza A/Duck/Singapore/97 (H5N3) vaccine: a potential priming strategy.* J Infect Dis, 2005. **191**(8): p. 1210-5.
7. Le, Q.M., et al., *Avian flu: isolation of drug-resistant H5N1 virus.* Nature, 2005. **437**(7062): p. 1108.
8. McMichael, A.J., et al., *Cytotoxic T-cell immunity to influenza.* N Engl J Med, 1983. **309**(1): p. 13-7.
9. Ulmer, J.B., et al., *Heterologous protection against influenza by injection of DNA encoding a viral protein.* Science, 1993. **259**(5102): p. 1745-9.
10. Epstein, S.L., et al., *Protection against multiple influenza A subtypes by vaccination with highly conserved nucleoprotein.* Vaccine, 2005.
11. McShane, H., et al., *Recombinant modified vaccinia virus Ankara expressing antigen 85A boosts BCG-primed and naturally acquired antimycobacterial immunity in humans.* Nat Med, 2004. **10**(11): p. 1240-4.
12. McConkey, S.J., et al., *Enhanced T-cell immunogenicity of plasmid DNA vaccines boosted by recombinant modified vaccinia virus Ankara in humans.* Nat Med, 2003. **9**(6): p. 729-35.
13. Webster, D.P., et al., *Enhanced T cell-mediated protection against malaria in human challenges by using the recombinant poxviruses FP9 and modified vaccinia virus Ankara.* Proc Natl Acad Sci U S A, 2005. **102**(13): p. 4836-41.
14. Blanchard, T.J., et al., *Modified vaccinia virus Ankara undergoes limited replication in human cells and lacks several immunomodulatory proteins: implications for use as a human vaccine.* J. Gen. Virol., 1998. **79**(Pt 5): p. 1159-67.
15. Mayr, A., et al., *The smallpox vaccination strain MVA: marker, genetic structure, experience gained with the parenteral vaccination and behavior in organisms with a debilitated defence mechanism.* Zentralbl. Bakteriol. [B], 1978. **167**(5-6): p. 375-90.
16. Sutter, G. and B. Moss, *Nonreplicating vaccinia vector efficiently expresses recombinant genes.* Proc. Natl. Acad. Sci., 1992. **89**(22): p. 10847-51.
17. Cosma, A., et al., *Therapeutic vaccination with MVA-HIV-1 nef elicits Nef-specific T-helper cell responses in chronically HIV-1 infected individuals.* Vaccine, 2003. **22**(1): p. 21-9.
18. Dorrell, L., et al., *Safety and tolerability of recombinant modified vaccinia virus Ankara expressing an HIV-1 gag/multiepitope immunogen (MVA.HIVA) in HIV-1-infected persons receiving combination antiretroviral therapy.* Vaccine, 2007. **25**(17): p. 3277-83.
19. Goonetilleke, N., et al., *Induction of multifunctional human immunodeficiency virus type 1 (HIV-1)-specific T cells capable of proliferation in healthy subjects by using a prime-boost regimen of DNA- and modified vaccinia virus Ankara-vectored vaccines expressing HIV-1 Gag coupled to CD8+ T-cell epitopes.* J Virol, 2006. **80**(10): p. 4717-28.

20. Bejon, P., et al., *Safety profile of the viral vectors of attenuated fowlpox strain FP9 and modified vaccinia virus Ankara recombinant for either of 2 preerythrocytic malaria antigens, ME-TRAP or the circumsporozoite protein, in children and adults in Kenya.* Clin Infect Dis, 2006. **42**(8): p. 1102-10.
21. Meyer, R.G., et al., *A phase I vaccination study with tyrosinase in patients with stage II melanoma using recombinant modified vaccinia virus Ankara (MVA-hTyr).* Cancer Immunol Immunother, 2005. **54**(5): p. 453-67.
22. Moorthy, V.S., et al., *Safety and immunogenicity of DNA/modified vaccinia virus ankara malaria vaccination in African adults.* J Infect Dis, 2003. **188**(8): p. 1239-44.
23. Smith, C.L., et al., *Recombinant modified vaccinia Ankara primes functionally activated CTL specific for a melanoma tumor antigen epitope in melanoma patients with a high risk of disease recurrence.* Int J Cancer, 2005. **113**(2): p. 259-66.
24. Webster, D.P., et al., *Safety of recombinant fowlpox strain FP9 and modified vaccinia virus Ankara vaccines against liver-stage P. falciparum malaria in non-immune volunteers.* Vaccine, 2006. **24**(15): p. 3026-34.
25. Dorrell, L., et al., *Safety and tolerability of recombinant modified vaccinia virus Ankara expressing an HIV-1 gag/multiepitope immunogen (MVA.HIVA) in HIV-1-infected persons receiving combination antiretroviral therapy.* Vaccine, 2007. **25**(17): p. 3277-83.
26. Peters, B.S., et al., *Studies of a prophylactic HIV-1 vaccine candidate based on modified vaccinia virus Ankara (MVA) with and without DNA priming: effects of dosage and route on safety and immunogenicity.* Vaccine, 2007. **25**(11): p. 2120-7.
